# Highly Dispersed Rhodium on MXenes via Microwave Solvothermal Strategy for High‐Performance Hydrogen Evolution Catalysis

**DOI:** 10.1002/smll.202510349

**Published:** 2025-12-19

**Authors:** Anton S. Zverev, Christopher Penschke, Leonardo Cancellara, Stefan Reinicke, Christina Günter, Sibylle Rüstig, Namitha Deepak, Sergio Kogikoski Jr, Peter Saalfrank, Ilko Bald

**Affiliations:** ^1^ Institute of Chemistry University of Potsdam 14476 Potsdam Germany; ^2^ Max Planck Institute of Colloids and Interfaces 14476 Potsdam Germany; ^3^ Fraunhofer Institute for Applied Polymer Research IAP 14476 Potsdam Germany; ^4^ Institute of Geosciences University of Potsdam 14476 Potsdam Germany; ^5^ Dynamics of Molecules and Clusters Department J. Heyrovský Institute of Physical Chemistry of the CAS Prague 18223 Czech Republic

**Keywords:** Ab Initio DFT modeling, electrocatalysis, hydrogen evolution reaction, microwave assisted solvothermal treatment, MXenes, rhodium catalytic sites

## Abstract

This work presents a novel microwave‐assisted solvothermal method for decorating nanoflakes of transition‐metal carbides (MXenes) Ti_3_C_2_ and V_2_C with highly dispersed rhodium catalytic sites, significantly enhancing the electrocatalytic efficiency of the hydrogen evolution reaction (HER). The results indicate that microwave treatment does not significantly alter the nanoflake structure but promotes the formation of subnanometer‐sized Rh catalytic sites. A combined analysis of density functional theory‐calculated core‐level shifts and experimental X‐ray photoelectron (XPS) spectra identifies the most likely structures of the Rh catalytic centers formed through the microwave‐assisted solvothermal process. Rh anchored to the oxygen‐terminated MXene nanoflake surface, bonded to two or three oxygen atoms (RhO_n_), explains the Rh 3d XPS band with a notable chemical shift. Rh‐decorated nanoflakes display superior catalytic performance in acidic, basic, and neutral media compared to pure MXenes. Turnover frequencies (TOF) suggest that the HER catalytic activity of Rh sites is comparable to or exceeds that of pure platinum surface atoms. Using rhodium catalytic site structures as an example, it is demonstrated that the mutual arrangement of the Gibbs free energy of hydrogen adsorption on the catalytic site, in cases with protonated and non‐protonated terminal groups of the nanoflake, can serve as a criterion for electrocatalytic efficiency.

## Graphical abstract

This study presents a microwave‐assisted method to decorate MXene nanoflakes with rhodium (Rh) subnanometer catalytic sites. The material's structure remains intact, while electrocatalytic activity improves across different pH conditions. Detailed experimental and computational analyses identify the structure of Rh catalytic sites, providing insight into catalyst design and the factors that govern electrocatalytic performance.

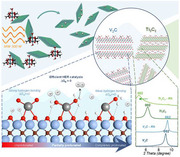

## 1 Introduction

Renewable energy has been the focus of scientific research and technological development for over half a century.^[^ ^1^, ^2^, ^3^, ^4^ ^]^ It has gained increasing relevance due to the growing and evident consequences of global warming,^[^ ^5^, ^6^, ^7^ ^]^ as well as the rising influence of the fuel market on global political stability.^[^ ^8^ ^]^ Hydrogen, despite its specific challenges in storage and transportation, is considered a key element in renewable technologies^[^ ^4^, ^9^, ^10^, ^11^ ^]^ due to the simplicity of its synthesis and the absence of harmful oxidation products. The development of efficient, reliable, and readily available electrocatalysts is a central task in advancing hydrogen technologies. This challenge can be addressed by searching for and developing new catalytic materials^[^ ^12^, ^13^, ^14^, ^15^ ^]^ or optimizing the architecture of highly efficient catalysts, such as platinum, rhodium, and iridium.^[^ ^12^, ^16^, ^17^, ^18^, ^19^, ^20^ ^]^ In the latter case, the ideal outcome is the atomically dispersed scaling of catalytic metals to maximize the activity of each atom.^[^ ^21^ ^]^ Various catalytic supports can be employed to solve this task and protect catalytic particles from aggregation.^[^ ^22^, ^23^ ^]^ Supports should have a high surface area, effectively anchor the catalyst to their surface, and, in the case of an electrocatalytic process, possess significant electrical conductivity. Ideally, the support should also function as a co‐catalyst, enhancing the activity of the primary catalytic center.^[^ ^24^, ^25^ ^]^

MXenes – 2D transition metal carbides and nitrides – perfectly satisfy all the requirements and can be applied as efficient support for single‐atom catalytic centers.^[^ ^26^, ^27^, ^28^, ^29^ ^]^ MXenes is a general term for 2D nanoflakes obtained by etching MAX‐phase ceramics, which consist of layers of transition metal (M) carbides or nitrides (X) separated by layers of more chemically active metals (A), such as aluminum.^[^ ^30^, ^31^ ^]^ After dissolving the A‐layer and delamination, few‐layer nanoflakes can reach sizes of several microns in planar dimensions and have a high surface area, which is covered by polar terminal groups, including halogen atoms (F, Cl) and oxygen groups (─OH, ─O─). This allows catalytic sites to be anchored onto them. MXenes are conductive materials and can be dispersed in water and polar solvents. MXenes exhibit electrocatalytic activity in the hydrogen evolution reaction, but it is limited by the high binding energy between protons and terminals.^[^ ^29^ ^]^

MXenes can easily oxidize,^[^ ^32^, ^33^ ^]^ especially when heated in either air or water. It makes the protection of the MXene nanoflakes from oxidation the main challenge at each step of the formation of electrocatalytically active sites on the MXene surface. To achieve sub‐nanosized dispersion of the catalyst, the metal precursor is typically adsorbed onto the MXene from an aqueous solution by freeze‐drying and then decomposed by high‐temperature heating the material in a constant flow of argon to protect the MXene nanoflakes from irreversible agglomeration and oxidation.^[^ ^34^, ^35^, ^36^ ^]^ Any manipulation with dry MXenes, such as grinding, also requires an inert atmosphere. This fact also makes it necessary to have precise control of the phase composition of the MXenes to manage the possible formation of the oxide phase via Raman spectroscopy and diffractometry.^[^ ^37^ ^]^

In this study, we present a strategy to avoid all the above work‐ and energy‐consuming procedures in synthesizing Rh‐decorated MXenes electrocatalysts for hydrogen evolution reaction (HER). We demonstrated the decoration of Ti_3_C_2_ and V_2_C MXenes with Rh catalytic sites through microwave‐assisted solvothermal synthesis, investigated the structure of the obtained materials, showing that the MXene nanoflakes remain almost intact, and experimentally and theoretically determined the impact of Rh on the MXenes' electrocatalytic efficiency in the hydrogen evolution reaction.

## 2 Results and Discussion

### 2.1 Composition and Structure of Pure and Rh‐Decorated MXenes

#### 2.1.1 Decoration of MXenes by Rhodium Catalytic Sites

Titanium carbide (Ti_3_C_2_) 2D nanoflakes were synthesized by etching of Ti_3_AlC_2_ in LiF‐HCl solution,^[^ ^38^ ^]^ while V_2_C nanoflakes were synthesized by etching of V_2_AlC in HF‐HCl mixture followed by delamination in 5% Tetrabutylammonium hydroxide (TBAOH) (Figure S1, Supporting Information).^[^ ^32^ ^]^ MXenes were modified with rhodium (Rh) catalytic sites via microwave‐driven solvothermal synthesis (Figure S2, Supporting Information). Briefly, MXenes were dispersed in a rhodium acetate/acetonitrile solution, and the resulting suspension was treated in a microwave synthesis station under 300 W microwave irradiation for 30 min (Figure S3, Supporting Information). The Rh‐decorated flakes were then washed and transferred to ethanol for further investigation. To prevent the oxidation of MXenes, a non‐aqueous and oxygen‐free solvent was used for solvothermal treatment. A key advantage of acetonitrile is its high transparency to microwave radiation,^[^ ^39^ ^]^ which allows the MXene nanoflakes to function as hot plates for rhodium acetate decomposition and facilitates the metal dispersion across the nanoflake surface. The approach applied in the current work is simpler, less energy‐intensive, and less expensive compared to previous methods, making it more suitable for widespread applications, e.g., in industry. It allows for the adherence to wet chemistry methods, working only with MXene colloidal solutions, which can be transferred through pipe systems, while avoiding energy‐consuming processes by using microwave radiation, which is becoming a widely used tool in the chemical industry.^[^ ^40^, ^41^, ^42^ ^]^

#### 2.1.2 Crystall Structure and Morphology of Materials

**Figure 1:**
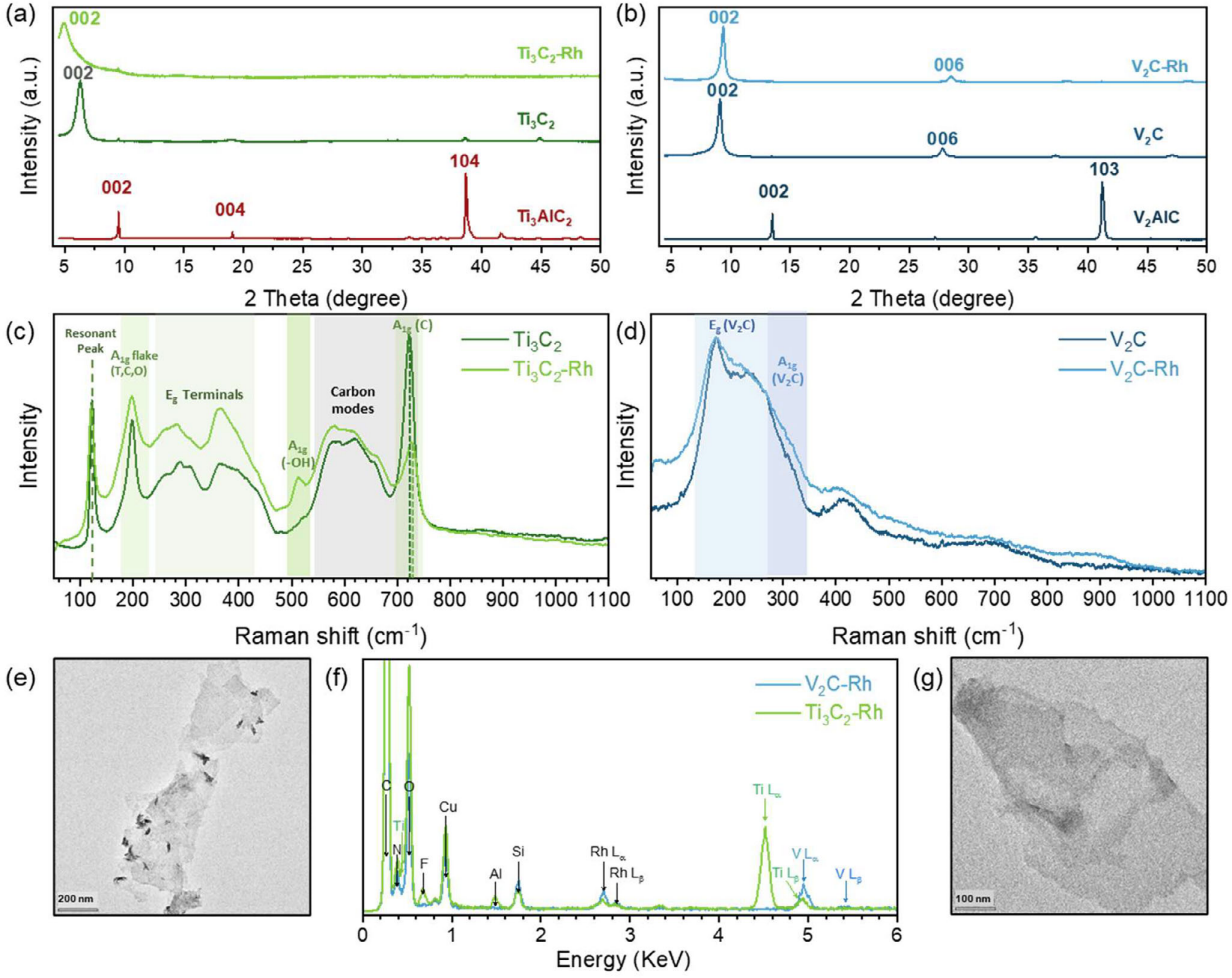
X‐ray diffractograms of Ti_3_AlC_2_, Ti_3_C_2_, Ti_3_C_2_‐Rh a) and V_2_AlC, V_2_C, V_2_C ‐Rh b); Raman spectra of Ti_3_C_2_, Ti_3_C_2_‐Rh c) and V_2_C, V_2_C ‐Rh d), TEM images of Ti_3_C_2_‐Rh e) and V_2_C ‐Rh g); Energy‐dispersive X‐ray spectra of Ti_3_C_2_‐Rh and V_2_C ‐Rh f).

X‐ray diffractograms (XRD) of all samples are shown in **Figure** **1**a,b, and interlayer distances, average flake thickness, and the number of MXene layers in the flakes, estimated using the Scherrer equation, are combined into Table S1 (Supporting Information). The peaks corresponding to 002 lattice planes become dominant and shift to smaller angles for both MXenes (Ti_3_C_2_ and V_2_C) compared to the MAX phases (Ti_3_AlC_2_ and V_2_AlC). This result indicates that both MXene samples are composed of few‐layer nanoflakes.^[^ ^32^, ^38^ ^]^ After microwave‐solvothermal treatment, no peaks corresponding to metallic rhodium or rhodium oxides were detected in the diffractograms. The treatment affects the 002‐interlayer distance of MXenes. In Ti_3_C_2_, this leads to a significant increase in interlayer spacing, up to 3.8 Å, and a decrease in the average number of layers per flake. It can be caused by changes in terminal group composition, but, due to the significance of the effect, the anchoring of the Rh sites to each side of the Ti_3_C_2_ layers seems more probable. In contrast, the position of the 002 peak of V_2_C shifted slightly to larger angles, indicating a decrease in interlayer distance, which can be caused by deleting TBAOH molecules, used as a delamination agent, from the interlayer space.

To evaluate potential oxidation during treatment, Raman spectra were collected using a 785 nm excitation laser (Figure 1c,d). The spectrum of Ti_3_C_2_ showed three intense peaks at 120, 196, and 720 cm^−1^, corresponding to the resonant, A_1g_(Ti, O, C) and A_1g_(C) modes. Two broader regions containing several vibrational modes between 230–470 cm^−1^ and 560–730 cm^−1^ relate to in‐plane (E_g_) vibrations of terminal groups and carbon, respectively. The last region also includes a band near 600 cm^−1^, associated with A_1g_ modes of oxygen terminals. This Raman spectral pattern is characteristic of Ti_3_C_2_ nanoflakes.^[^ ^43^ ^]^ For Ti_3_C_2_‐Rh, changes were observed in the intensity ratios of the main peaks, along with a shift in the maximum of the A_1g_(C) mode from 720 to 726 cm^−1^. This shift may indicate a change in the composition of terminal groups from halogen atoms to oxygen groups, as well as a variation in the number of Ti_3_C_2_ layers. The peak at 515 cm^−1^, related to the A_1g_(‐OH) vibration, becomes more prominent in Ti_3_C_2_‐Rh, which is also reported to reflect alterations in terminal group composition.^[^ ^43^ ^]^ The Raman spectra of V_2_C and V_2_C‐Rh are essentially identical, featuring a strong, non‐elementary peak with two maxima ≈175 and 230–235 cm^−1^, corresponding to the E_g_ and A_1g_ vibrational modes of the flake, as cited in the literature.^[^ ^44^ ^]^ Less intense peaks at 417, 534, and 685 cm^−1^ are associated with combined in‐plane and out‐of‐plane vibrational modes of the terminals. No additional peaks indicative of oxidation products, such as vanadium or titanium oxides, or rhodium phases, were detected in either Ti_3_C_2_ or the less stable V_2_C MXenes after solvothermal treatment

TEM images are shown in Figure 1e,g and Figure S4 (Supporting Information). All the samples consist of nanoflakes with planar sizes of several hundred nanometers, as well as agglomerates of these flakes. Large multilayered V_2_C flakes exhibit porous structures, while Ti_3_C_2_ flakes contain visible particles formed by rumpled MXene layers (Figure 1e). No significant differences were observed in the morphology of the flakes before and after the treatment. We did not detect any particles corresponding to another crystalline phase of metal Rh or rhodium oxides on the surface of the MXenes flakes. However, the energy‐dispersive X‐ray (EDX) spectra of Ti_3_C_2_‐Rh and V_2_C‐Rh confirm the presence of rhodium in both samples (Figure 1f).

**Figure 2:**
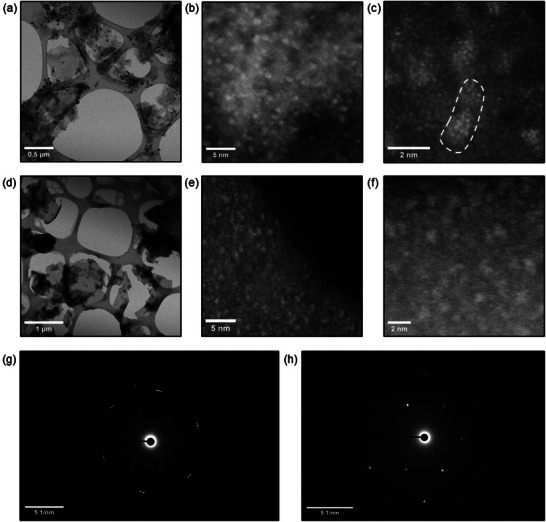
Bright‐field STEM overview images of Ti_3_C_2_‐Rh a) and V_2_C ‐Rh d) nanoflakes; HAADF‐STEM images of Ti_3_C_2_‐Rh b,c) and V_2_C ‐Rh e,f); Selected area diffractograms Ti_3_C_2_‐Rh g) and V_2_C ‐Rh h) nanoflakes.

High‐Angle Annular Dark Field Scanning Transmission Electron Microscopy (HAADF‐STEM) images of rhodium sites on the surface of Ti_3_C_2_ (**Figure** **2**b,c) and V_2_C (Figure 2e,f) demonstrate that the MXene nanoflakes are covered with an amorphous layer, which makes the MXene lattice hardly visible. On the surfaces of both types of nanoflakes, contrast areas associated with Rh sites (0.5–1.5 nm) were distinguished. In the case of the Ti_3_C_2_‐Rh sample, the Rh was dispersed as island structures with diameters of 1–1.5 nm, consisting of smaller fragments of ≈0.15 nm, which we can associate with individual Rh atoms (Figure 2c). The Rh sites on V_2_C are roughly several times larger, ≈0,4‐1 nm, and can be represented as small clusters of a few Rh atoms.

Selected area electron diffractograms of Rh‐decorated Ti_3_C_2_ (Figure 2g) and V_2_C (Figure 2h) demonstrate diffraction reflexes of (101) and (110) crystal planes. The d‐spacings of the Ti_3_C_2_‐Rh sample are 0.2623 nm for the (101) plane and 0.1596 nm for the (101) plane, resulting in a lattice parameter a of 3.071 Å. The a‐parameter of the V_2_C lattice of the V_2_C‐Rh sample of 2.915 Å was calculated using the d‐spacing of 0.2527 nm and 0.1453 nm for the (101) and (110) planes, respectively. The obtained lattice parameters are in good agreement with literature data.^[^ ^45^, ^46^ ^]^

#### 2.1.3 X‐Ray Photoelectron Spectroscopy of the Samples

**Figure 3:**
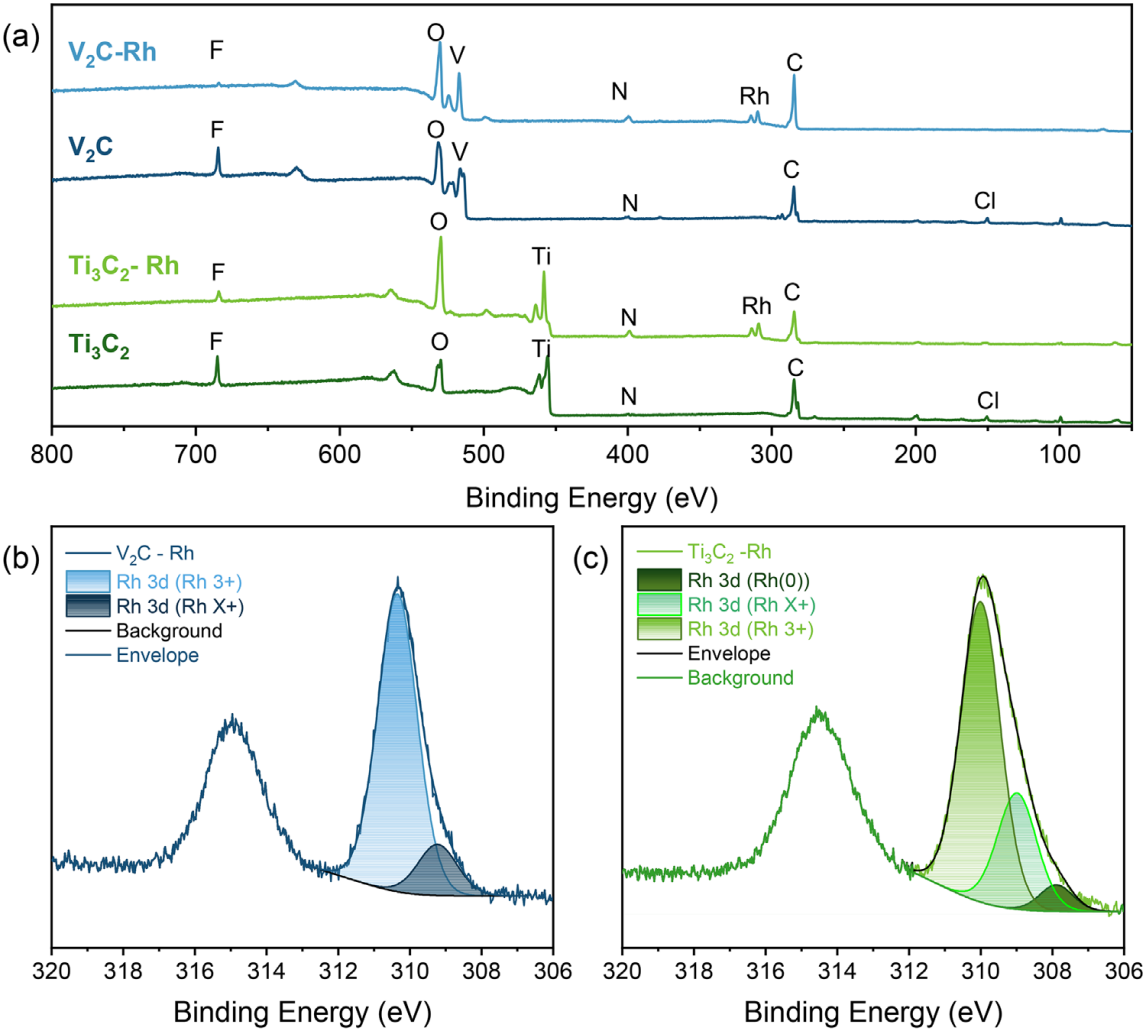
X‐ray photoelectron spectra of pure and Rh‐modified MXenes a) Detailed X‐ray photoelectron spectra of Rh 3d peak of V_2_C ‐Rh b) and Ti_3_C_2_‐Rh c) with approximation of Rh 3d_5/2_ peak by the set of Gaussians.

**Figure** **3**a shows X‐ray photoelectron spectra (XPS) of the pristine and Rh‐decorated samples, and the atomic percentages of elements, calculated using XPS spectra, are presented in Table S2 (Supporting Information). Oxygen concentrations increased in both materials after the treatment, while the concentration of halogen atoms decreased. This can help Rh to anchor to the nanoflakes' surface. The increase in nitrogen concentration in both samples suggests possible anchoring of acetonitrile to the flakes’ surface. Changing of the intensity ratios of the transition metal bands shows some transferring of Ti^3+^ (456 eV) and V^3+^(514 eV) to Ti^4+^(458 eV) and V^5+^(517–518 eV), respectively. These changes can indicate partial oxidation of the nanoflakes' surfaces, explaining the presence of an amorphous layer not detected by other methods. Rhodium was reliably detected in both samples, with atomic percentages of ≈0.89% and 1.43% for V_2_C and Ti_3_C_2_, respectively, resulting in Rh‐to‐titanium or vanadium ratios of 0.1 and 0.025, respectively. This difference can be explained by the difference in the average thickness of MXene nanoflakes. According to estimation via the Scherrer equation, the Ti_3_C_2_‐Rh sample represented by relatively thin nanoflakes of 4‐5 layers (Table S1, Supporting Information), and V_2_C‐Rh has an average number of layers more than 20, resulting in a thickness of the flake of ≈21 nm, which is higher than the penetration depth accessible with XPS investigation.

The Rh 3d_5/2_ peak can be deconvoluted into three Gaussian modes in the case of Ti_3_C_2_‐Rh and two modes in V_2_C‐Rh (Figure 3b,c). The lowest energy band at 307.6 eV, which can be associated with metallic Rh^0^ sites, was detected only in the Ti_3_C_2_‐Rh sample. However, the intensity of this band is significantly lower compared to other bands. The highest energy mode with a maximum at 309.9 eV in Ti_3_C_2_‐Rh and 310.3 eV in V_2_C ‐Rh dominates in both samples significantly over the modes at 308.9 and 309.2 eV, respectively. The lower energy bands (308.9 ± 0.4 eV) can be attributed to Rh^3^⁺, corresponding to Rh_2_O_3_.^[^ ^47^ ^]^ Interpretation of higher energy bands is a more complicated task. The 310 ± 0.2 eV band generally corresponds to RhCl_3_; however, this explanation seems unlikely due to the extremely low chlorine content on the flakes’ surface. A band with a maximum of ≈309.4–309.5 eV corresponds to Rh^4+^ in RhO_2_.^[^ ^48^, ^49^ ^]^ However, the position of the maxima found in Rh‐decorated MXenes is still higher than the typical position of Rh^4+^ states. A similar shift of the Rh 3d peak to higher binding energies has been observed in Rh‐SA decorated catalysts.^[^ ^50^, ^51^, ^52^ ^]^ The shift to higher energies has also been reported for Rh_2_O_3_ dispersed into atomically distributed (single‐atom) Rh^3^⁺ sites.^[^ ^51^, ^52^ ^]^

The Rh 3d core‐level shifts of several possible Rh catalytic sites were calculated via density functional theory (DFT) method using periodic supercell models (computational details are given in the Methods section below). Except for the rhodium atom anchored to the oxygen terminals, we considered the Rh site with one, two, and three bonded oxygen atoms (Tables S3 and Figures S8–S11, Supporting Information, as well as Section 2.3 below), designated here and below as MXene‐RhO, MXene‐RhO_2_, MXene‐RhO_3_, and MXene‐RhO_n_ in the general case (not to be confused with rhodium oxides). The sequential oxygen adsorption energies for these structures, indicating the probability of occurrence and high stability of these structures, are presented. Table S4 (Supporting Information) contains the calculated Rh 3d core‐level shifts for Rh catalytic centers considered in Table S3 (Supporting Information), as well as Rh (III) and (IV) oxides, with respect to bulk Rh. These data were calculated for protonated (MXene surface covered with hydroxyls) and nonprotonated (MXene surface covered with oxygens) conditions, and absolute values differ for these two cases. Nevertheless, the increase of 3d core‐level shifts with increasing number of bonded oxygens is obvious for all kinds of systems. It allows us to assign the dominant XPS peaks at ≈310 eV to more complex Rh sites with additional oxygen atoms.

#### 2.1.4 Effects of Rh Decoration on Materials Structure and Composition

Therefore, we can conclude that solvothermal microwave‐assisted treatment enables the decoration of Ti_3_C_2_ and V_2_C nanoflakes with Rh catalytic sites while preserving the structure of the MXenes, without significant oxidation of the material.

The morphology of Ti_3_C_2_ nanoflakes underwent significant changes following solvothermal treatment. The interlayer distance of Ti_3_C_2_ increased by ≈1–3.8 Å, while the average number of layers per flake decreased by nearly half. This effect cannot be attributed solely to changes in the composition of terminal groups, as similar changes in terminal group composition were observed in both samples. We propose that the observed morphological changes may result from the deposition of Rh sites across the entire Ti_3_C_2_ surface, including the internal layers within the flakes.

The difference in response to the treatment can be explained by variations in the intrinsic properties of the MXenes. The initial interlayer spacing of Ti_3_C_2_ is noticeably larger than that of V_2_C, making its surface more accessible for modification, including the anchoring of Rh sites. This also explains the higher Rh concentration observed in Ti_3_C_2_‐Rh samples.

Microwave solvothermal treatment results in partial oxidation of the nanoflake surface, forming an amorphous layer in which the island structures of rhodium sites can be distinguished. In the case of Ti_3_C_2_‐Rh, it is possible to see the Rh atoms constructing the sites. By combining morphology with XPS data and calculated results, we can infer that Rh sites are mainly represented by islands of compact, disordered atoms or small subnanometer clusters with a rhodium oxide (RhO_n_) chemical structure.

### 2.2 Evaluation of the HER Electrocatalytic Performance

**Figure 4:**
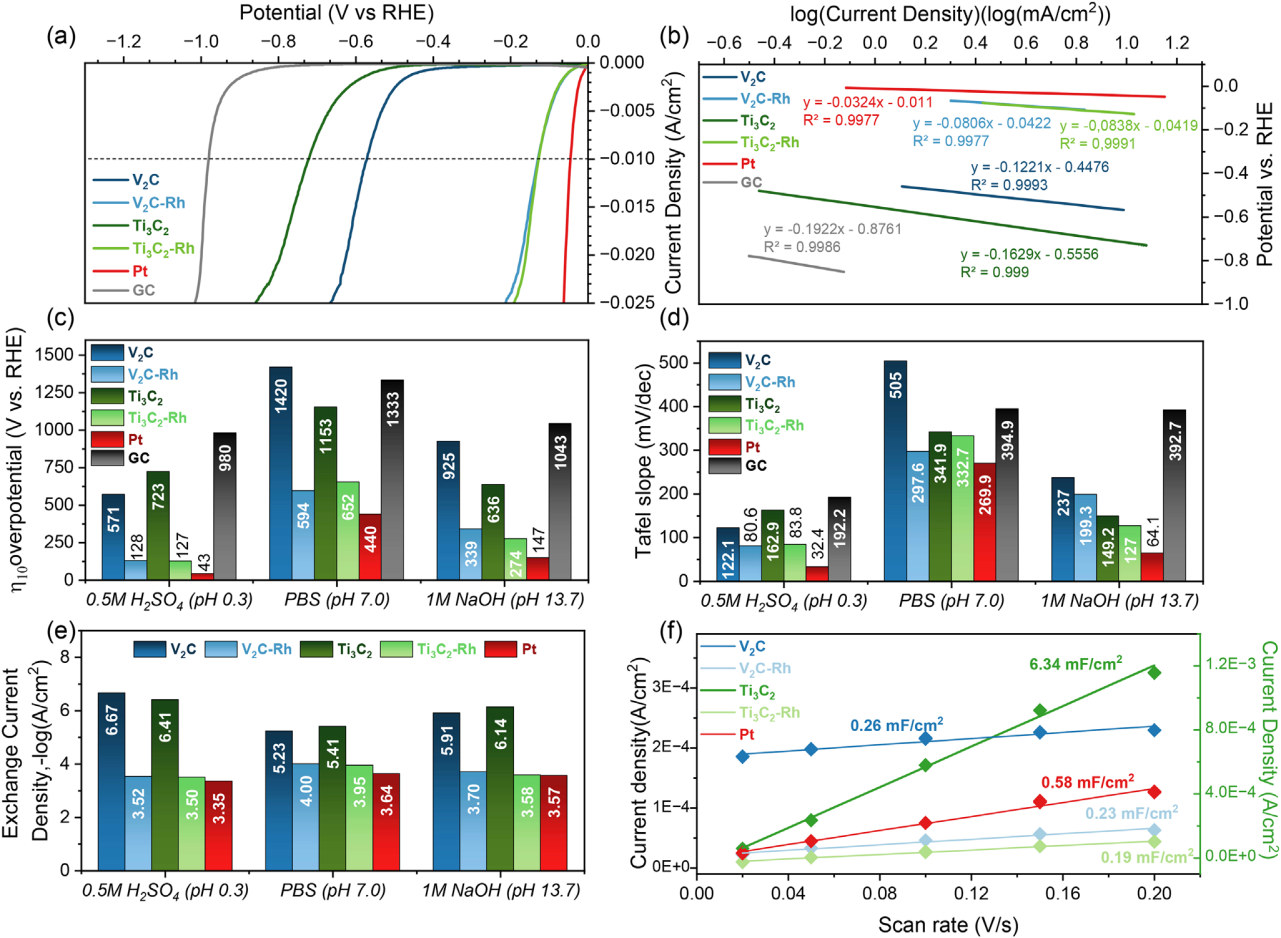
Electrochemical evaluation of the obtained electrocatalysts and reference materials towards HER. a) LSV curve for HER in 0.5 m H_2_SO_4_; b) Linear fitting of Tafel plots for HER in 0.5 m H_2_SO_4_; c) Comparison of η_10_ for all tested samples (Figure S5a,b, Supporting Information); d) Comparison of Tafel slopes (Figure S5b,d, Supporting Information) (e); Comparison of the negative logarithms of the exchange current densities; g) Linear fits of capacitive currents versus CV scan rates. The left axis corresponds to the Ti_3_C_2_ values.

**Figure** **4**a presents the linear sweep polarization (LSP) curves of pure and Rh‐decorated MXenes deposited on a glassy carbon electrode (GCE, d = 5 mm) in comparison to a platinum electrode (d = 2 mm) in an acidic medium (0.5 m H_2_SO_4_, pH = 0.3). Figure 4c shows the overpotentials required to achieve a current density of 10 mA cm^−^^2^ (η_10_) in acidic, neutral (PBS, pH = 7.0), and basic (1 m NaOH, pH = 13.7) media, compared with the Pt electrode and an empty GC electrode.

In acidic media, the platinum electrode exhibits η_10_ = 43 mV, while in basic and neutral media, it has η_10_ values of 147 and 440 mV, respectively. Pure Ti_3_C_2_ demonstrates clear electrocatalytic activity in all three tested media. In 1N acid and base solutions, it exhibits η_10_ values of 723 and 636 mV, respectively, which correspond well to the literature data.^[^ ^53^, ^54^ ^]^ In the neutral solution, its η_10_ (1153 mV) is slightly lower than that of the GC electrode. V_2_C nanoflakes exhibit higher electrocatalytic activity in acidic media but show no significant activity in basic media. At pH = 7, the bare GCE has a lower η_10_ (1333 mV) than the V_2_C‐coated electrode (>1400 mV). Moreover, the V_2_C film begins to degrade after several HER cycles. Rhodium decoration significantly enhances the electrocatalytic activity of MXenes in all tested solutions, yielding relatively similar overpotentials in 0.5 m H_2_SO_4_; the η_10_ values for Ti_3_C_2_‐Rh and V_2_C‐Rh are 127 and 128 mV, respectively. In 1 m NaOH, Ti_3_C_2_‐Rh exhibits a lower η_10_ (274 mV) than V_2_C‐Rh (339 mV). However, in the neutral electrolyte, V_2_C‐Rh achieves a lower overpotential (594 mV) than Ti_3_C_2_‐Rh (652 mV), despite the electrocatalytic inactivity of pure V_2_C nanoflakes.

Figure 4b and Figure S5b,d (Supporting Information) present the Tafel plots for all polarization curves. Using these data, we calculated the Tafel slopes (Figure 4d) and exchange current densities (Figure 4e) for each sample to compare their catalytic activity with one another and with the Pt electrode. The ratio of Tafel slopes generally follows the same trend as the overpotentials at 10 mA cm^−^^2^ (η_10_). The exchange current density values provide a clearer indication of the enhancement in electrocatalytic activity. In acidic and basic conditions, the values for rhodium‐decorated MXenes are more than two orders of magnitude higher than those of the pure MXenes. The exchange current densities of the Rh‐decorated MXenes are relatively close to those of platinum. The largest difference, about a factor of two, was observed in the PBS solution. In contrast, in basic conditions, the values for Ti_3_C_2_‐Rh and platinum are nearly identical.

The double‐layer capacitances (Figure 4f) of the materials were estimated using the capacitance current plots (Figure S7, Supporting Information) and were 0.26 and 0.23 mF cm^−^^2^ for V_2_C and V_2_C‐Rh, 6.34 and 0.19 mF cm^−^^2^ for Ti_3_C_2_ and Ti_3_C_2_‐Rh, and 0.58 mF cm^−^^2^ for Pt, respectively.

**Figure 5:**
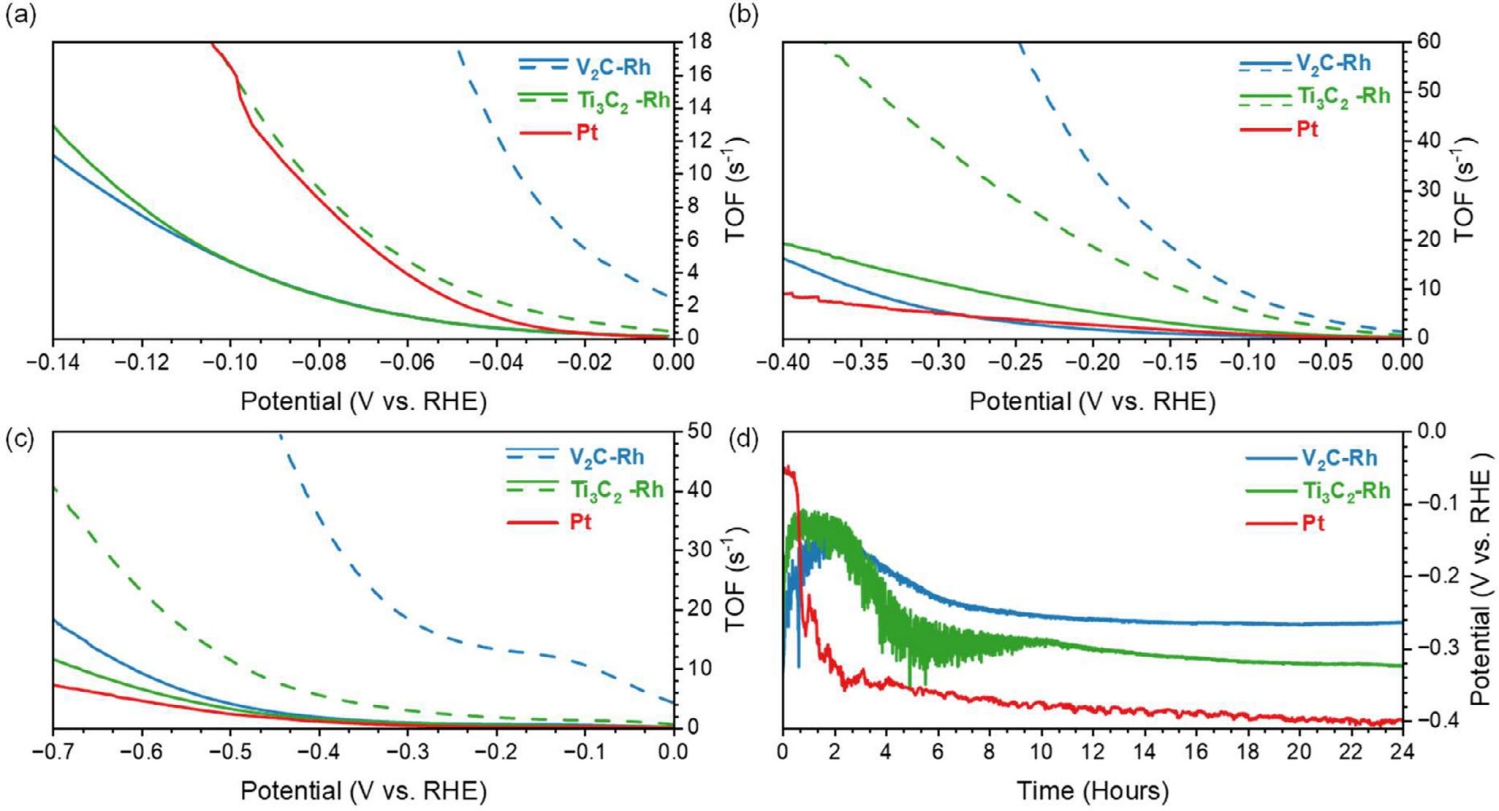
TOF of the Rh‐decorated MXenes and Pt as a function of overpotential in 0.5 m H_2_SO_4_ a) 1 m NaOH b), and PBS c) without (dashed lines) and with (solid lines) taking the average number of layers in a flake into account. Stability test of HER electrocatalytic activity of materials. Chronopotentiometry curves of rhodium‐decorated MXenes and platinum electrode in 0.5  H_2_SO_4_ d).

The turnover frequency (TOF) for each LSV measurement was estimated based on electrochemically active surface area, Rh concentration based on XPS studies, and the number of layers in the MXene flakes. Here, we would like to underline that the TOF calculation is one of the most complicated tasks of catalytic studies.^[^ ^55^ ^]^ Since the number of catalytic sites participating in the reaction is specifically challenging to assess, it requires numerous assumptions, and any derived values are, a priori, significantly speculative and should be considered only as an estimate. In **Figure** **5**a–c, two sets of TOF as a function of overpotential, calculated with different assumptions, are presented for each Rh‐decorated MXene. In the case of solid lines, we considered that Rh can decorate only the surfaces of flakes but not the surfaces of inner MXene layers (see Equations (3) and (4) in the Experimental Section). Dashed lines are calculated according to the hypothesis that Rh can cover the whole surface of MXene, without the factor *N*, i.e., the average number of layers in the flake (see Equations (3) and (4) in the Experimental Section).

As the interlayer distance does not increase for V_2_C ‐Rh upon Rh decoration (Table S1, Supporting Information), we don't have any reason to consider that Rh can decorate the inner surfaces of MXene layers. Therefore, the curves calculated without including the average number of layers are likely to be significantly overestimated. In the case of Ti_3_C_2_‐Rh, there is a notable increase in the interlayer distance after treatment, indicating that the Rh sites could cover nearly the entire surface of the MXene layers, including the inner layers of the flake. According to this point of view, the TOF of Ti_3_C_2_‐Rh in acid media should be closer to platinum values (16.5 s^−1^ at 0.1 V) than to the V_2_C‐Rh ones (4.7 s^−1^ at 0.1 V). The TOF values of the Rh‐decorated MXenes calculated according to both assumptions are equal to or higher than the Pt values in basic and neutral media. If we calculate an analogue of TOF using exchange current densities (to take into account the number of catalytic sites), all the MXene‐Rh values will be higher than the platinum ones. Comparing two Rh‐decorated MXenes, we can assume that the electrocatalytic activity of Rh sites on the Ti_3_C_2_ flakes should be higher than on the V_2_C.

Comparison of the HER performance parameters of the obtained Rh‐decorated electrocatalysts is presented in Table S5 (Supporting Information). The TOF values listed in Table S5 (Supporting Information) show considerable variation; however, the estimated V_2_C‐Rh and Ti_3_C_2_‐Rh TOF values are at least of the same order of magnitude. The only material that directly corresponds to Ti_3_C_2_‐Rh has a similar TOF value.^[^ ^34^ ^]^

To investigate electrocatalytic stability, chronopotentiometry curves of the Rh‐decorated samples and the platinum electrode were recorded at a current density of 10 mA cm^−^^2^ in acidic medium, where the catalysts show the highest activity (Figure 5d). All the curves exhibit significant noise due to shielding of the catalyst surface by hydrogen bubbles, which notably affects the shape of the MXene samples' curves. Both Rh‐decorated MXene samples exhibit better stability than the pure platinum surface. While the decline in catalytic efficiency of the platinum electrode can be attributed to surface poisoning by oxidation, interaction with sulfur derivatives, or intercalated hydrogen, the changes in the catalytic activity of MXenes can be explained by morphological changes in the MXene film due to the physical impact of hydrogen bubble formation. Initially, this process increases the availability of Rh sites, but over time, it leads to film degradation. We observed a progressive increase in the electrocatalytic activity of both pure and Rh‐decorated MXenes in line‐sweep voltammetry (LSV) tests, as indicated by a shift of the polarization curve towards lower potentials over several cycles until a stable and reproducible shape was reached. And the minimal overpotential reached on chronopotentiometry curves well corresponds to LSV data.

### 2.3 Computational Studies of the Electrocatalytic Sites' Performance

**Figure 6:**
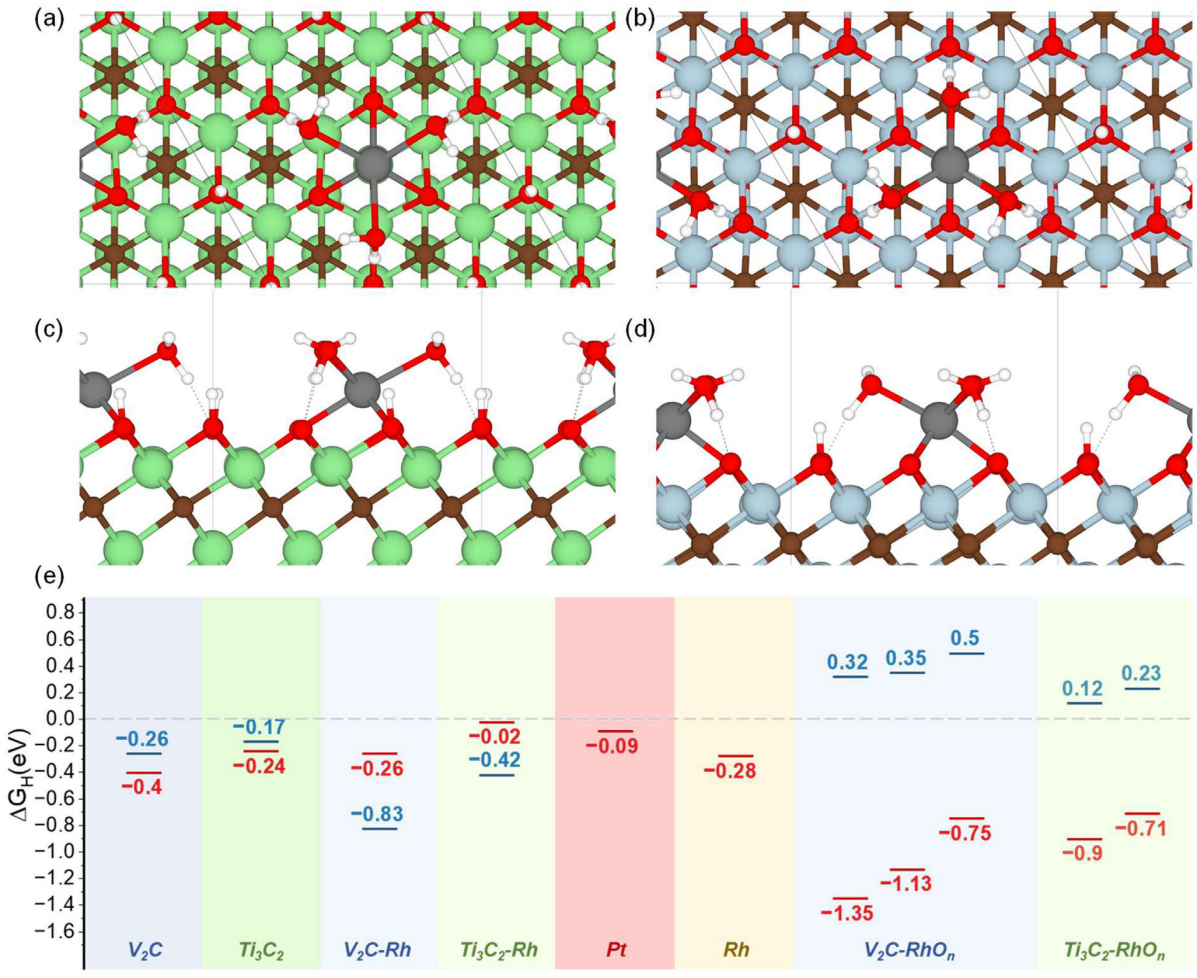
Top and side of the Rh site on the protonated a) Ti_3_C_2_ and b) V_2_C. V ‐ blue; Ti ‐ green; C ‐ brown; O ‐ red; H ‐ white; Rh ‐ grey. c) Gibbs free energy of hydrogen adsorption on the pure MXenes’ surface, Rh atom (without oxygen) anchored on the MXene surface, metal Pt and Rh, and several examples of RhO_n_ sites (see Table S3, Supporting Information). Red solid lines correspond to nonprotonated conditions, and blue dashed lines correspond to protonated conditions.

The Gibbs free energy (*∆G_H_*) of the hydrogen atom adsorption on the pristine MXenes surface and Rh catalytic sites was calculated via the density functional theory method (details are given in the Methods section below). This value is typically used as a reactivity descriptor within Nørskov's computational hydrogen electrode (CHE) model.^[^ ^56^ ^]^ Too weak adsorption (high positive *∆G_H_*) hinders the first step of the HER, while too strong adsorption (high negative *∆G_H_*) makes the second step, the removal of H to form a hydrogen molecule, energetically unfavorable. Within the CHE model, the Gibbs free energy of hydrogen atom adsorption directly correlates to the overpotential required for the HER. The MXenes covered by only oxygen terminals were used as a model system. Two limiting cases representing a wholly hydrogen‐free surface and a wholly protonated surface were considered. Several possible structures of Rh catalytic sites have been used in calculations: Rh single atom, and more complex Rh sites (RhO_n_) with additional oxygen atoms, hydroxyl groups, or coordinated water molecules, represented in **Figure** **6**a,b, and Figures S8–S11 (Supporting Information). The high number of possible compositions (i.e, how many O and H atoms are added) together with the large structural flexibility of the adsorbed RhO_n_ species leads to a complex phase space; however, not all the considered model systems are formally possible. Bonding of three oxygen atoms to the rhodium atom anchored to the nonprotonated Ti_3_C_2_ and V_2_C surface leads to the detachment of the complex (Figure S11, Supporting Information). However, on the protonated surfaces, the Rh atom remains bonded to the terminal oxygen of MXenes, stabilized by proton transfer to the three oxygen atoms, which converts them into hydroxyl groups.

Comparison of the Gibbs free energy of hydrogen adsorption (Figure 6c) during the transition from nonprotonated to wholly protonated conditions of the same model system reveals a significant change in values, with these changes differing both qualitatively and quantitatively between the considered systems. Gibbs free energy of hydrogen adsorption on the protonated pure Ti_3_C_2_ and V_2_C surface is closer to zero (−0.17 and −0.26 eV, respectively) than on the nonprotonated ones (−0.24 and −0.4 eV, respectively). For the Rh atom (without bonded oxygen atoms), we observed the opposite phenomenon. In the nonprotonated case, hydrogen adsorption on oxygen atoms is energetically preferred compared to adsorption on Rh. The situation completely changes for protonated MXenes, for which Rh shows high hydrogen adsorption energies (Figure 6c). All the RhO_n_ sites anchored to the nonprotonated MXene surface strongly bind hydrogen, with adsorption energies typically ≈‐1 eV. This indicates that they are unlikely to serve as active sites for hydrogen evolution. However, in the protonated state, the situation changes dramatically: adsorption energies move closer to zero, and some become positive, and the latter case is the most intriguing.

Although the calculations represent only two limiting cases, if the *∆G_H_* changes its sign between these cases, they suggest that adsorption at some intermediate protonation levels should become close to thermoneutral, leading to highly active HER catalytic sites. An optimal protonation level of the MXene surface, providing hydrogen adsorption Gibbs free energy of about zero, was demonstrated theoretically for the Mo_2_C pure surface,^[^ ^57^ ^]^ the MXene with the highest HER electrocatalytic activity. The protonation is also not a static characteristic and changes during the HER process, as experimentally confirmed for Ti_3_C_2_ by operando Raman^[^ ^35^, ^58^ ^]^ and FTIR^[^ ^59^ ^]^ spectroelectrochemistry. It allows us to assume that changing the sign of the *∆G_H_* from negative to positive, for nonprotonated and protonated cases, serves as a criterion of high electrocatalytic activity of the system, indicating that the system can have *∆G_H_* ~0, even if its *∆G_H_* values in the two limiting cases are far from zero.

The Rh atom anchored to the Ti_3_C_2_ oxygen‐covered nonprotonated surface, the simplest and most obvious model system often considered in calculations,^[^ ^34^ ^]^ shows the closest to zero hydrogen adsorption energy of −0.02 eV. However, in the protonated condition, this energy decreases to −0.42 eV, which is even lower than that of pure MXenes. It is also important to mention that the single standing metal atom bonded to the MXene surface should not be stable in real conditions, which is supported by highly negative adsorption energies of oxygens to the Rh atom (Table S3, Supporting Information).

The Rh catalytic sites with two and three bonded oxygen atoms allow us to simultaneously explain the high electrocatalytic activity of the Rh‐decorated MXenes and the XPS Rh 3d band with an unusually far shift to high energies. These sites have low energies of formation (oxygen adsorption), high 3d core‐level shifts, and correspond to the proposed criterion of different *∆G_H_* sign for protonated and nonprotonated conditions. Therefore, these sites can be possible candidates for the catalytic sites observed in the experiment.

## 3 Conclusion

**Figure 7:**
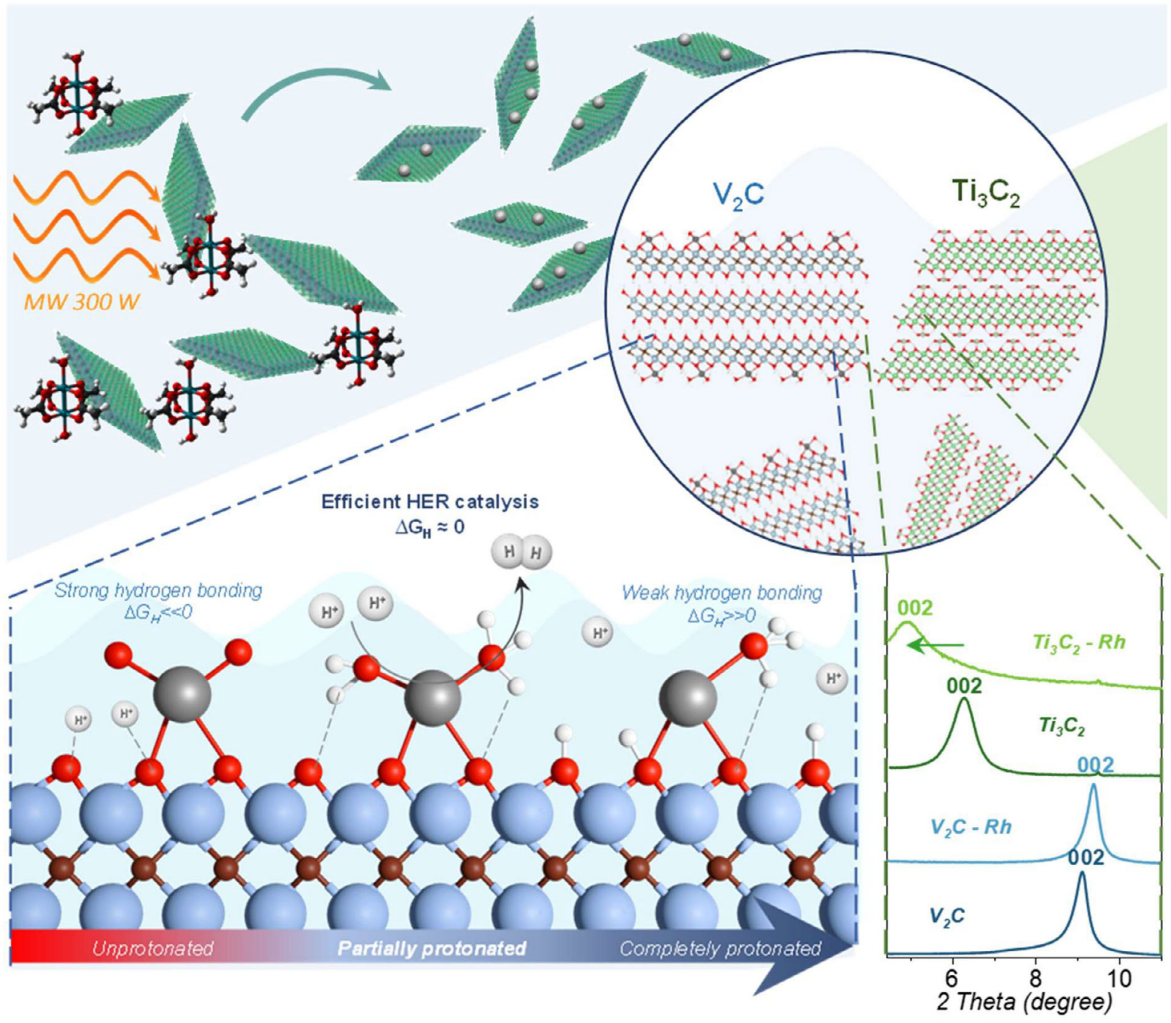
Schematic of MXene decoration with the rhodium catalytic sites and hydrogen evolution reaction on them.

Ti_3_C_2_ and V_2_C MXenes were successfully decorated with highly dispersed rhodium catalytic sites via microwave‐assisted solvothermal treatment, while maintaining their initial structure, even in the case of the significantly less stable V_2_C nanoflakes. Rh‐catalytic sites are represented by subnanometer islands or clusters of disordered rhodium oxide species. We propose that the localization of rhodium catalytic sites differs for each of the MXenes: in Ti_3_C_2_, Rh sites are distributed across the surfaces of individual MXene layers, leading to a significant increase in interlayer distance. In contrast, in V_2_C, rhodium appears to deposit only on the external surfaces of the flakes, with limited penetration into inner layers (**Figure** **7**). This observation can also account for the higher rhodium concentration in Ti_3_C_2_ (≈1.5 at.%) compared to V_2_C (≈0.8 at.%).

X‐ray photoelectron spectroscopy (XPS) data suggest that only a small fraction of rhodium in the Ti_3_C_2_–Rh sample can be attributed to the Rh° form. Rh^3^⁺ species are present in both samples at slightly higher concentrations. DFT modeling of the Rh catalytic sites demonstrates an increase in the 3d core‐level shifts with increasing number of oxygen atoms bonded to the Rh and explains the dominant spectral peak in both samples with a maximum ≈109.9–110.3 eV.

The proposed MXene modification strategy offers several advantages critical for industrial applications. It eliminates the need to process MXenes in solid, dried form, enabling treatment exclusively in nanoflake suspensions. This approach facilitates transport via pipelines and allows for the design of a continuous catalyst production process. The resulting suspension can be directly used for electrode fabrication by depositing it onto a conductive substrate with a high surface area.

The decoration of MXene flakes with Rh significantly enhances their electrocatalytic activity across acidic, neutral, and basic media. The Rh‐modified samples exhibit markedly lower overpotentials at 10 mA cm^−2^ and higher exchange current densities compared to the pristine MXenes. A comparison of turnover frequencies (TOFs) reveals that Rh‐decorated Ti_3_C_2_ exhibits superior catalytic activity compared to V_2_C–Rh, approaching that of platinum in acidic media and surpassing it in basic and neutral environments. Although the TOF of V_2_C–Rh is ≈3 times lower than that of surface Pt atoms in acidic media, it remains comparable or superior in neutral and basic conditions. It was shown that considering the actual morphology of MXenes (the average number of MXene layers in the flake) is necessary to estimate the concentration of catalytic sites accurately. Both Rh‐modified MXenes exhibit greater electrocatalytic stability than platinum.

DFT calculated Gibbs free energies of hydrogen adsorption in two critical conditions, completely protonated and completely nonprotonated MXene nanoflake surface, were proposed as a criterion of electrocatalytic efficiency of the catalytic site. If the catalytic site on the nonprotonated surface has a negative *∆G_H_* and on the protonated surface a positive one, it signals to us that this site has *∆G_H_* ≈0 at some protonation level and can be electrocatalytically active (Figure 7). Protonation of the nanoflakes' surface increases with increasing overpotential and enhances the HER activity when *∆G_H_* comes closer to zero. This criterion was applied to all the possible catalytic sites, and it is met only for Rh centers bonded with oxygen atoms (RhO_n_), which makes them probable catalytic sites in the obtained materials.

## 4 Experimental Section

### Synthesis of MXenes

Titanium carbide (Ti_3_C_2_) 2D nanoflakes were synthesized using the MILD method.^[^ ^38^ ^]^ Lithium fluoride (Merck) (800 mg) was dissolved in 10 mL of 9 m HCl (AnalaR NORMAPUR^®^) in a 20 mL polyethylene vial. The solution was stirred at 400 rpm for 30 min, after which 0.5 g of Ti_3_AlC_2_ MAX‐phase (Nanografi, Turkey) was slowly added to the solution. Once the vigorous hydrogen release subsided, the vial remained unsealed and was kept at room temperature under continuous stirring at 400 rpm for 24 h. Subsequently, the mixture was transferred to a 50 mL centrifuge tube containing Millipore water and centrifuged at 2520 rcf. After centrifugation, the supernatant was carefully removed using a pipette and replaced with fresh water. The material was then redispersed by a combination of vigorous hand shaking and vortex stirring at 1000 rpm for 1 min. The washing procedure was repeated with an increased centrifugation speed of 5000 rcf to minimize product loss until the pH approached 6. Subsequent washing steps were performed at 2520 rcf, and the supernatant, containing the Ti_3_C_2_ nanoflake suspension, was collected. This process was repeated until the supernatant became nearly transparent. For the final washing steps, Millipore water saturated with argon was used, with argon continuously bubbled through the collected solution during the procedure. The prepared suspension was stored in a refrigerator.

V_2_C nanoflakes were synthesized following the method.^[^ ^32^ ^]^ V_2_AlC MAX‐phase (Nanografi, Turkey) (1 g) was etched in a mixture of 8 mL of 12 m HCl (AnalaR NORMAPUR^®^) and 12 mL of 48% HF (AnalaR NORMAPUR^®^) for 96 h. The resulting suspension was transferred to a 50 mL centrifuge tube containing Millipore water and centrifuged at 2520 rcf. The washing procedure was then repeated until the pH of the supernatant reached ≈6. The obtained MXene slurry was immediately transferred to a 50 mL conical flask containing 20 mL argon‐ saturated Millipore water with 2.5 mL 40% TBAOH solution in methanol (Alfa Aesar™). The suspension was stirred at 400 rpm for 20 h under an argon atmosphere. The prepared material was collected through washing with argon‐saturated Millipore water, followed by centrifugation at 2520 rcf. Throughout the entire collection process, argon‐saturated water was used, and suspensions were continuously saturated with argon. The final suspension was stored in a refrigerator.

### Microwave‐Assisted Decoration of MXene Nanoflakes by Rhodium Catalytic Sites

The Ti_3_C_2_ and V_2_C nanoflakes were transferred from an aqueous medium to acetonitrile. In the first stage, the water suspension was centrifuged at 14 × g for 35 min, the supernatant was replaced with ethanol (AnalaR NORMAPUR), and the procedure was repeated using acetonitrile (99.5%, Sigma‐Aldrich). Acetonitrile was then removed by centrifugation, and the MXene was redispersed in fresh acetonitrile to prepare a suspension with a concentration of ≈3 mg mL^−1^. The MXene suspension (6 mL, ≈1 mg mL^−1^) was mixed with a rhodium acetate solution in acetonitrile and pure acetonitrile. The Rh/MXene molar ratio in the mixture for microwave‐assisted synthesis was ≈1:15. Synthesis was performed using a CEM Discover SP microwave synthesis station (CEM Corporation) equipped with 10 mL tube reactors. The reaction was carried out under microwave irradiation at 300 W for 30 min. During the process, the solution temperature reached 190–191 °C, and the pressure initially increased to 215 PSI before decreasing to 170–175 PSI (Figure S3, Supporting Information). The product was washed twice with ethanol and then dispersed into 2 mL of pure ethanol to obtain an MXene suspension with a concentration of 3 mg mL^−1^.

### X‐Ray Diffractometry

Diffractograms of the initial MAX‐phase powders, pure MXenes, and Rh‐decorated samples were obtained using an Empyrean diffractometer (Malvern Panalytical, Netherlands) equipped with a Cu X‐ray source. Films of pristine and Rh‐decorated MXenes were prepared by drop‐casting ethanol suspensions onto a background‐free silicon plate. The average flake thickness and the average number of layers per flake were estimated via the Scherrer equation (Table S1, Supporting Information).

### Transmission Electron Microscopy

The structure of the samples was examined using transmission electron microscopy (TEM) using a JEOL JEM‐1011 microscope (JEOL, Japan) and FEI Talos F200S transmission electron microscope operated at 200 kV. To prepare the samples, a 30 µL droplet of nanoparticle suspension was placed onto a copper grid and left to dry naturally at ambient temperature. Elemental composition analysis was performed using energy‐dispersive X‐ray spectroscopy (EDX), equipped with two silicon drift detectors (SDD), to ensure accurate detection of sample constituents.

### High‐Resolution Transmission Electron Microscopy

The samples dispersed in ethanol were sonicated for several minutes and drop‐casted on a lacey carbon grid for TEM observations. The (scanning) transmission electron micrographs were acquired using a JEOL^TM^ARM200F with a cold field‐emission gun and double aberration correction by CEOS GmbH, operated at 80 kV with an emission current of 10 µA. TEM and electron diffraction images were taken using a Gatan^TM^ OneView CMOS camera. High‐angle annular dark field (HAADF)‐STEM micrographs were acquired using an electron probe with a nominal convergence semi‐angle of 68 mrad, and a JEOL^TM^annular detector with inner and outer acceptance angles of 50 and 180 mrad, respectively.

*Raman measurements* were performed using a LabRam HR Evolution Raman Microscope (HORIBA, France). For the measurements, an MXene film was prepared on a silicon wafer by drop‐casting from an ethanol suspension. The spectra were recorded using a 785 nm excitation laser and a 100× objective lens, providing a power fluence of 77 kW cm^−^^2^ over an area of 0.89 µm^2^.

*XPS measurements* were performed on an AXIS Supra+ instrument (Kratos Analytical, U.K.). For this purpose, monochromatic Al Kα‐radiation (300 W) was used for excitation. The instrument was operated in hybrid mode with electrostatic and magnetic lenses. For neutralization of the sample charges, thermal electrons from a filament were used. During the measurement, the instrument was operated with a take‐off angle of 90° with an analysis depth of ≈10 nm. Element identification, quantification, as well as formal fitting and charge shift correction were done with the software CasaXPS. For peak fitting, Gaussian functions were used. Samples were transferred to the XPS device and allowed to degas overnight. For each sample, measurements were performed at three different spots to account for surface inhomogeneities. For each spot, a survey spectrum for element identification and quantification was recorded, as well as O1s, C1s, and Rh3d fine spectra when dealing with a rhodium‐decorated sample. In the first round of measurements, samples were not electrically insulated from the sample holder. In the second round, insulating adhesive tape was used.

### Electrochemical Measurements

A typical three‐electrode cell was used for all electrochemical measurements. A glassy carbon (GC) electrode with the Teflon ring (BAS inc., Japan) (diameter of 5 mm), a graphite rod, and a saturated Ag/AgCl electrode were used as the working, counter, and reference electrodes, respectively. Phosphate buffer solution (PBS) at pH 7 (AVS TITRINORM, VWR), 0.5 m sulfuric acid prepared from 96% H_2_SO_4_ (Suprapur, Merck), and 1 m sodium hydroxide (EP, Merck) were used as electrolytes. A Metrohm Autolab PGSTAT302N potentiostat (Metrohm, Switzerland) was used for voltammogram recording. The working electrodes were prepared as follows: 500 µL of the catalyst solution in ethanol was centrifuged, and the solvent was removed. Then, 480 µL of pure ethanol and 20 µL of a 5% Nafion solution were added to the sediment. The resulting mixture was sonicated for 10 min and drop‐cast onto the GC electrode. To achieve a catalyst surface concentration of ≈1 mg cm^−^^2^, the solution was drop‐cast in three steps, with 10 µL applied in each step. The potential versus the reversible hydrogen electrode (RHE) was calculated using Equation (1). The differences between RHE and the saturated silver chloride electrode for acidic (pH = 0.301), basic (pH = 13.73), and neutral (pH = 7) conditions were 0.215, 1.009, and 0.611 V, respectively.

(1) $$E\left(\mathrm{RHE}\right)=E\left(\mathrm{Ag}/\mathrm{AgCl}\right)+0.197+0.0591\mathrm{pH}$$

*Linear sweep voltammetry* curves were measured with a step 0.0024 V and a scan rate of 0.01 Vs^−1^.

*Electrochemical impedance spectroscopy* (EIS) measurements were performed using the same sample as that used for LSV measurements, in the frequency range of 0.1 to 10⁵ Hz, with an amplitude of 0.01 V at the overpotential corresponding to a current density of 10 mA cm^−2^. All measured spectra closely matched the equivalent circuit shown in Figure S6a (Supporting Information). The value of R_1_ was used for iR drop correction.

*The double‐layer capacitance* was estimated using cyclic voltammetry in the potential range of 0.005–0.505 V versus RHE at various scan rates of 0.02, 0.05, 0.1, 0.15, and 0.2 V s^−1^ (Figure S7, Supporting Information). The capacitive current was calculated as the current density difference (*Δj/2*) at 0.255 V. The double‐layer capacitance was determined from the slope of the capacitive current versus scan rate (Figure 4e).

### TOF Calculation

TOF of the Rh catalytic centers and surface Pt atoms of the platinum electrode was estimated based on electrochemically active surface area, Rh concentration based on XPS studies, and the number of layers in the MXene flakes. Electrochemically active surface area (ECSA) can be estimated as a ratio of double‐layer capacitance (*C_dl_*) and specific capacitance (*C_s_*). *C_s_* was usually taken as 20–50 µF cm^−2^ in different studies. Here, the value of 40 µF cm^−2^ was used. Let us assume that, in the case of a platinum electrode, each platinum atom on the electrode surface acts as a catalytic site. Platinum has a face‐centered cubic lattice structure with a lattice parameter of a = 3.92 Å. By dividing the electrochemical surface area by the area of the unit cell face (*S_L_*) and multiplying by the number of atoms per unit cell face (which was 2 atoms for platinum), an estimate of the number of catalytic sites was obtained. Further dividing this value by Avogadro's number gives the concentration of platinum atoms per unit geometric area of the electrode (ϑ_*M*_). A similar calculation was applied for MXenes, considering their trigonal unit cell, which contains 1 atom per planar face, with a lattice parameter of a = 2.88 Å for V_2_C and a = 3.03 Å for Ti_3_C_2_, respectively. This allows us to estimate the number of catalytic sites for pure MXenes, assuming that each catalytic center corresponds to a surface transition metal atom. Next, it was assumed that the hydrogen evolution reaction occurs exclusively at rhodium catalytic sites, with each site corresponding to a rhodium atom. It was also assumed that rhodium atoms were dispersed on the surface of MXene nanosheets in such a way that all of them participate in the reaction. The concentration of rhodium atoms was determined based on its (*w_Rh_*) ratio to the surface transition metal atoms (*w_M_*), as obtained from X‐ray photoelectron spectroscopy analysis (Table S2, Supporting Information). For Ti_3_C_2_, it was taken into account that only 2 out of the 3 Ti atoms were located on the surface. This calculation assumes that the entire surface of each layer was available for rhodium decoration. Table S1 (Supporting Information) presents the number of layers in the MXene nanoflake. If it was assumed that the internal surfaces of the layers were inaccessible for decoration, then to correctly account for the concentration of rhodium atoms, the previously obtained expression was multiplied by N, which is the average number of layers per nanoflake. These final expressions (3, 4) provide the concentration of rhodium atoms per unit geometric surface area of the electrode, taking into account the actual morphology of the material (ϑ_*Rh*_). *TOF* of HER can be estimated using formula 5, where *i* was the current density, *F* – Faraday number.

(2) $$\vartheta_{M}=\frac{C_{dl}n_{L}}{C_{S}S_{L}N_{A}}$$

(3)

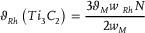

(4) $$\vartheta_{Rh}\left(V_{2}C\right)=\frac{\vartheta_{M}w_{Rh}N}{w_{M}}$$

(5) $$TOF=\frac{i}{2\vartheta_{Rh}F}$$

### Computational Details

All calculations were performed using VASP,^[^ ^60^, ^61^ ^]^ version 5.4.4. PAW pseudopotentials contained 12, 13, and 15 valence electrons for Ti, V, and Rh, respectively.^[^ ^62^, ^63^ ^]^ The PBE functional^[^ ^64^ ^]^ was employed together with the D3 dispersion correction^[^ ^65^ ^]^ with Becke‐Johnson damping.^[^ ^66^ ^]^ The plane wave energy cutoff was set to 600 eV. Spin‐polarized calculations were performed. The SCF and force convergence criteria were set to 10^−6^ eV and 10^−2^ eV Å^−1^, respectively. Lattice parameters were optimized using bulk models, resulting in values of *a_0_* = 3.03 Å and *a_0_* = 2.90 Å for Ti_3_C_2_ and V_2_C, respectively. Single‐layer models were used with a lattice vector of 20 Å along the surface normal to avoid interactions between periodic images. A supercell consisting of 3x3 primitive cells was employed, and the Brillouin zone was sampled with a 5x5x1 k‐point mesh. The bulk phases of Pt and Rh were optimized using 15x15x15 k‐point meshes, resulting in lattice constants of a_0_ = 3.93 Å and a_0_ = 3.79 Å for Pt and Rh, respectively. For the Pt(111) and Rh(111) surface models, 5x5 supercells with three layers were employed together with a 2x2x1 k‐point mesh. Gibbs free energies of hydrogen adsorption were calculated based on the reaction *+0.5H_2_ → *H, where * refers to the catalyst surface. All values refer to a temperature of 298.15 K and 1 atm pressure. Solvent effects were not considered. The hydrogen molecule was calculated as an isolated gas‐phase system. The Gibbs free energies contained all rotational and vibrational contributions of the hydrogen molecule, while only vibrational contributions were considered for the adsorbed species. Harmonic vibrational frequencies were obtained numerically using central differences with a displacement of 1.5 pm. Bader charges^[^ ^67^ ^]^ were calculated using a grid‐based approach.^[^ ^68^ ^]^ Images of structures have been created using VESTA.^[^ ^69^ ^]^

## Conflict of Interest

The authors declare no conflict of interest.

## Supporting information



Supporting Information

## Data Availability

The data that support the findings of this study are available from the corresponding author upon reasonable request
