# Supplementary material for: Highly Dispersed Rhodium on MXenes via Microwave Solvothermal Strategy for High‐Performance Hydrogen Evolution Catalysis
Source: Small. 2025 Dec 19;22(3):e10349. doi: 10.1002/smll.202510349 (PMC12802550; doi:10.1002/smll.202510349)
Supplement: Supplementary file 1 — Supporting Information [file SMLL-22-e10349-s001.docx]

Supporting Information

Highly dispersed rhodium on MXenes via microwave solvothermal strategy for high-performance hydrogen evolution catalysis

Anton S. Zverev*, Christopher Penschke, Leonardo Cancellara, Stefan Reinicke, Christina Günter, Sibylle Rüstig, Namitha Deepak, Sergio Kogikoski Jr., Peter Saalfrank, Ilko Bald


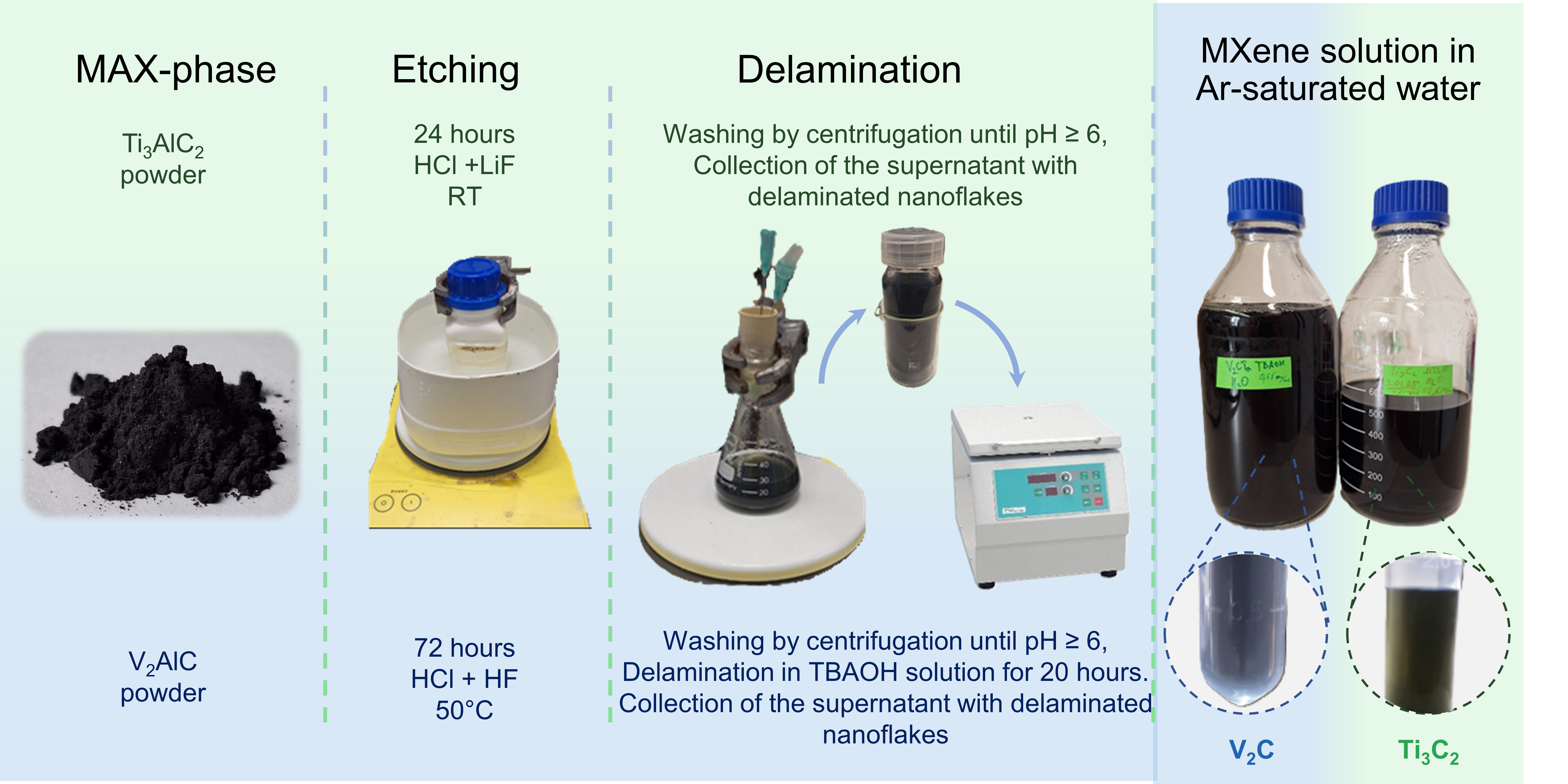


**Figure S1.** General scheme of the MXenes synthesis.


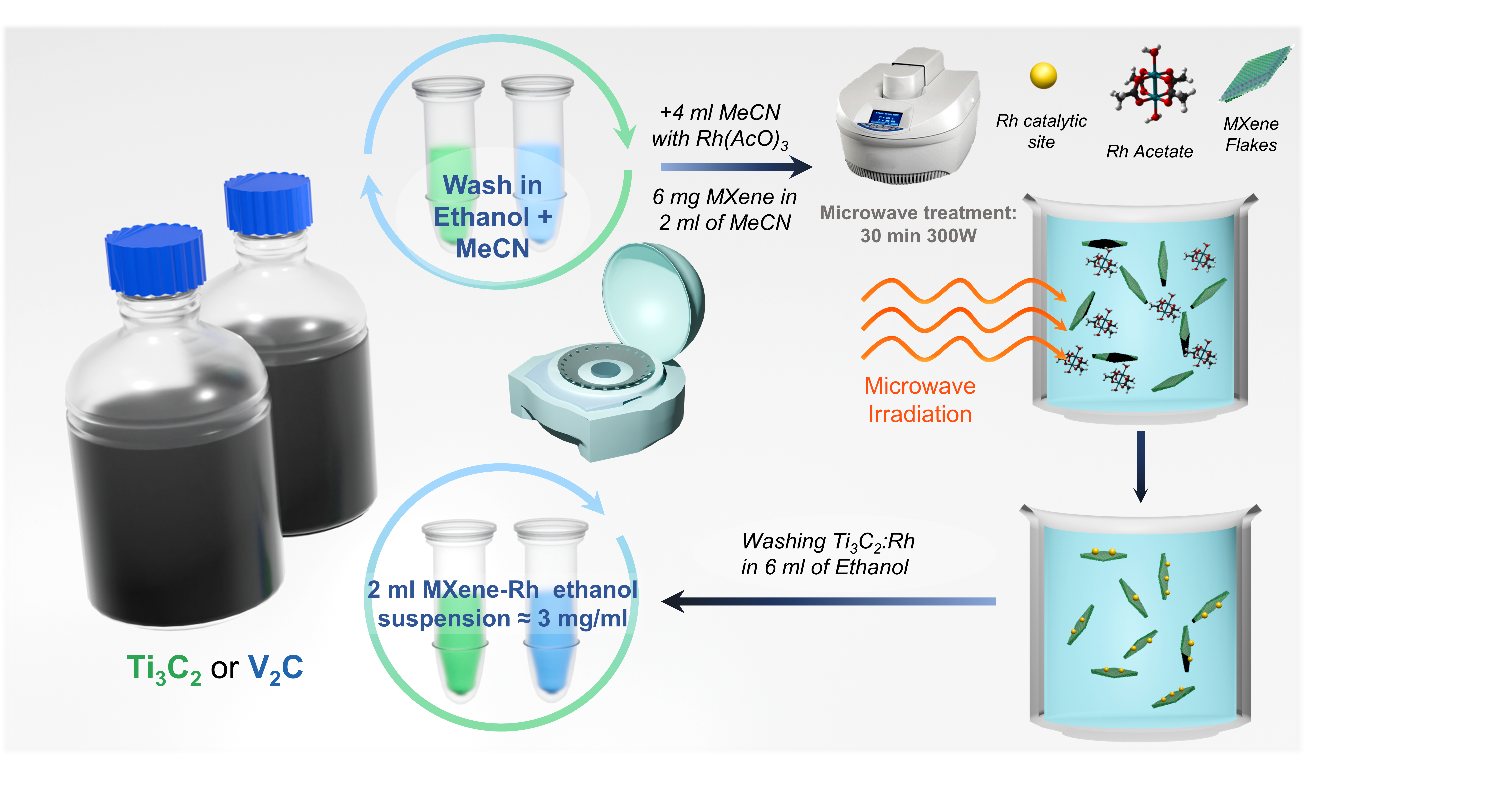


**Figure S2.** General scheme of Rh catalytic sites formation on MXene via microwave-assisted solvothermal synthesis.


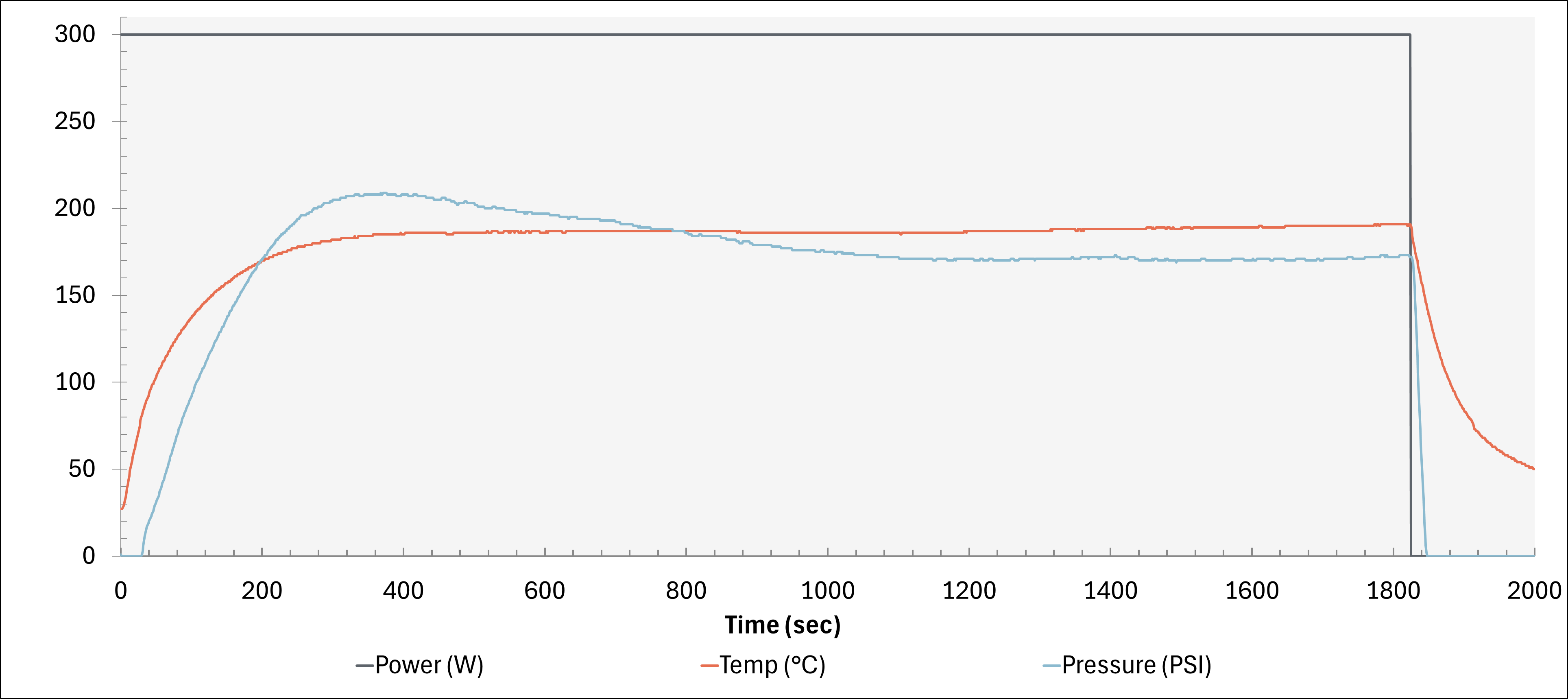


**Figure S3.** Microwave radiation power, temperature, and pressure as a function of time during the microwave-assisted solvothermal synthesis.


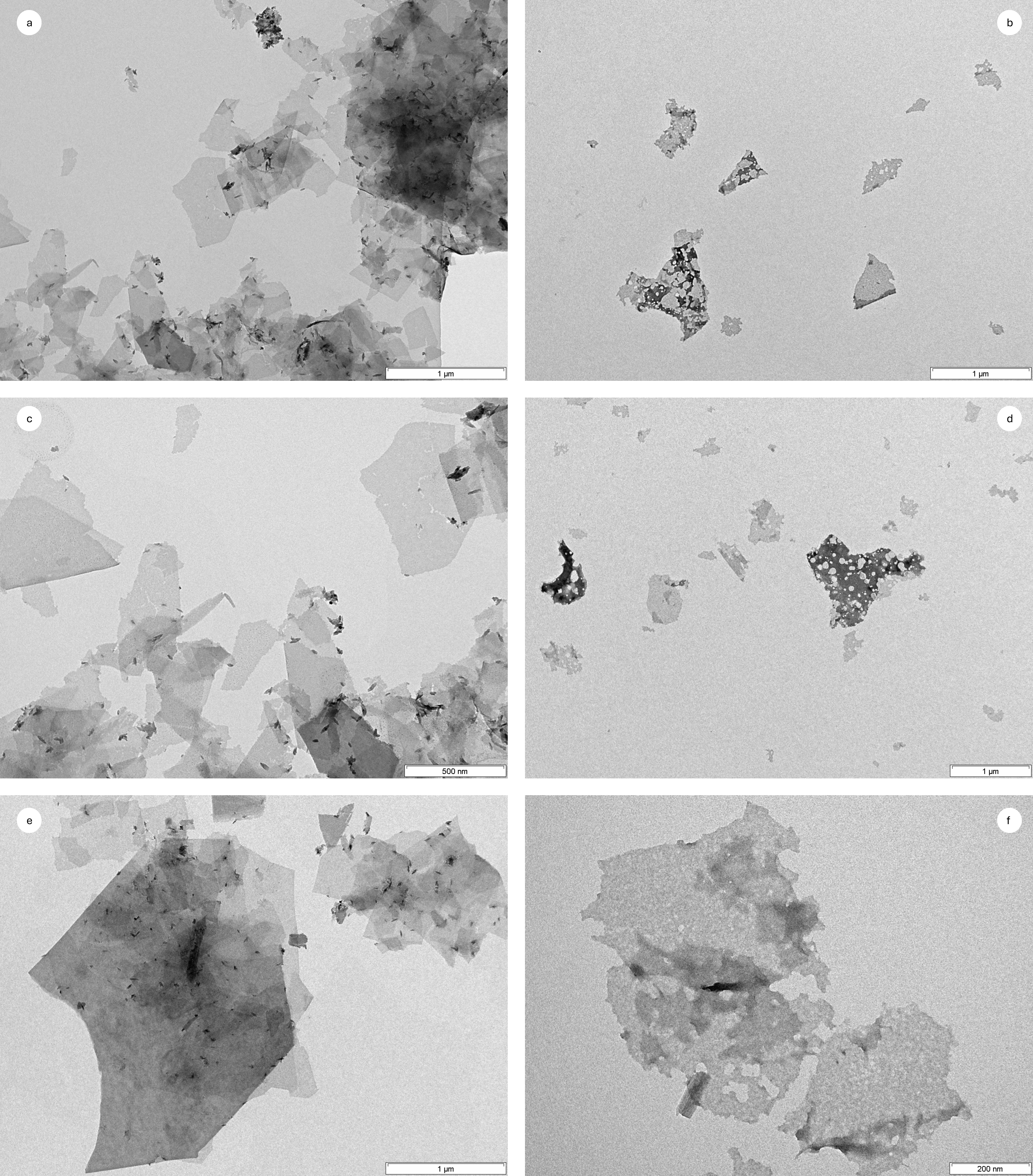


**Figure S4.** TEM images of the nanoflakes of Ti₃C₂-Rh (a, c, e) and V₂C-Rh (b, d, f).

**Table S1.** Estimation of average thickness and number of layers in nanoflakes, where *h* is the average thickness of the flake, and *w* is the percentage of flakes with the current morphology.

| Material | ***2Θ*_max_ [°]** | HWHM **[°]** | *d*(002) [Å] | ***h*** [Å] | Number of layers | *w* [%] |
| --- | --- | --- | --- | --- | --- | --- |
| V₂C | 9.115 | 0.153 | 9.694 | 262.875 | 28 | 44.7 |
|  | 8.983 | 0.168 | 9.836 | 238.595 | 25.3 | 6.8 |
|  | 8.791 | 0.463 | 10.051 | 86.414 | 9.5 | 30.6 |
|  | 7.806 | 0.926 | 11.317 | 43.076 | 4.8 | 17.8 |
| V₂C Rh | 9.351 | 0.186 | 9.450 | 215.728 | 23.8 | 100.0 |
| Ti₃C₂ | 6.268 | 0.324 | 14.089 | 123.300 | 9.8 | 100.0 |
| Ti₃C₂-Rh | 4.928 | 0.495 | 17.917 | 80.520 | 5.5 | 75.9 |
|  | 5.799 | 0.925 | 15.227 | 43.077 | 3.8 | 24.1 |

**Table S2.** Atomic Concentration [%] of the elements in pure and Rh-decorated MXenes’ nanoflakes.

| Material | Al 2p | C 1s | Cl 2p | F 1s | N 1s | O 1s | Rh 3d | Ti 2p | V 2p |
| --- | --- | --- | --- | --- | --- | --- | --- | --- | --- |
| V₂C -Rh | - | 40.99 | 0.18 | 0.40 | 3.06 | 18.69 | 0.79 | - | 35.90 |
| Ti₃C₂-Rh | 1.76 | 44.24 | 0.71 | 2.04 | 3.86 | 32.46 | 1.43 | 13.49 | - |
| V₂C | - | 26.38 | 0.24 | 2.45 | 0.72 | 13.83 | - | - | 56.36 |
| Ti₃C₂ | 1.07 | 51.91 | 1.60 | 5.53 | 0.92 | 20.27 | - | 18.68 | - |

**Table S3.** Sequential oxygen adsorption energies *∆E_ads_(O)* (MX-RhO_n-1_ + 0,5O_2_ → MX-RhO_n_) and sequential reaction free energies for hydrogen adsorption *∆G_H_* in eV (MX-RhO_n-1_H_m-1_ + 0,5O_2_ → MX-RhO_n_H_m_).

| System | ∆E_ads_(O) | *∆G_H_* | | | | | | |
| --- | --- | --- | --- | --- | --- | --- | --- | --- |
|  |  | 1st H | 2nd H | 3d H | 4th H | 5th H | 6th H | 7th H |
| V₂C -O | - | -0.40 | - | - | - | - | - | - |
| V₂C -O+Rh | - | -0.26 | - | - | - | - | - | - |
| V₂C -O+RhO | -1.30 | -1.09 | **-0.72** | **-0.26** | - | - | - | - |
| V₂C -O+RhO_2_ | -0.71 | -1.13 | **-1.35** | **-1.13** | **-0.75** | -0.38 | - | - |
| V₂C -O+RhO_3_ | -0.50 | **-1.47** | -1.22 | **-1.33** | -1.18 | -0.98 | -0.65 | -0.05 |
| V₂C -OH | - | -0.26 | - | - | - | - | - | - |
| V₂C -OH+Rh | - | -0.83 | - | - | - | - | - | - |
| V₂C -OH+ RhO | -3.63 | -0.68 | **+0.29** | **+0.32** | - | - | - | - |
| V₂C -OH+ RhO_2_ | -4.06 | -0.48 | **+0.32** | **+0.35** | **+0.50** | - | - | - |
| V₂C -OH+ RhO_3_ | -3.09 | **+0.02** | -0.38 | **+0.11** | **+0.33** | - | - | - |
| Ti₃C₂-O | - | -0.24 | - | - | - | - | - | - |
| Ti₃C₂-O+Rh | - | -0.02 | - | - | - | - | - | - |
| Ti₃C₂-O+RhO | -0.60 | -1.70 | -0.64 | **-0.40** | **-** | - | - | - |
| Ti₃C₂-O+ RhO_2_ | -2.08 | -0.77 | -0.53 | **-0.90** | **-0.71** | -0.09 | - | - |
| Ti₃C₂-O+ RhO_3_ | -0.22 | -1.43 | -0.74 | **-1.42** | **-1.03** | -0.72 | -0.33 | +0.03 |
| Ti₃C₂-OH | - | -0.17 | - | - | - | - | - | - |
| Ti₃C₂-OH+Rh | - | -0.42 | - | - | - | - | - | - |
| Ti₃C₂-OH+RhO | -3.28 | -0.43 | -1.06 | **+0.26** | - | - | - | - |
| Ti₃C₂-OH+ RhO_2_ | -4.01 | -0.27 | -0.82 | **+0.12** | **+0.23** | - | - | - |
| Ti₃C₂-OH+ RhO_3_ | -2.72 | -0.03 | -0.40 | **+0.09** | **+0.36** | - | - | - |

**Table S4.** Bader charges *q* and 3d core-level shifts *∆ε* compared to bulk Rh.

| System | V₂C | | | Ti₃C₂ | |
| --- | --- | --- | --- | --- | --- |
|  | *q* | *∆ε* | *q* | | *∆ε* |
| Rhodium (III) oxide Rh_2_O_3_ | 1.28 | 0.69 | 1.28 | | 0.69 |
| Rhodium (IV) oxide RhO_2_ | 1.58 | 1.93 | 1.58 | | 1.93 |
| MXene-O+Rh | 0.79 | -2.00 | 0.66 | | -2.13 |
| MXene-O+RhO | 1.30 | 0.73 | 1.17 | | 0.33 |
| MXene-O+RhO_2_ | 1.45 | 1.54 | 1.40 | | 2.36 |
| MXene-O+RhO_3_ | 1.59 | 2.31 | 1.53 | | 2.91 |
| MXene-OH+Rh | 0.27 | -2.21 | 0.06 | | -1.22 |
| MXene-OH+RhO | 0.70 | -0.16 | 0.64 | | -0.45 |
| MXene-OH+RhO_2_ | 0.92 | -0.83 | 0.52 | | -1.38 |
| MXene-OH+RhO_3_ | 1.21 | 0.28 | 1.21 | | 0.32 |


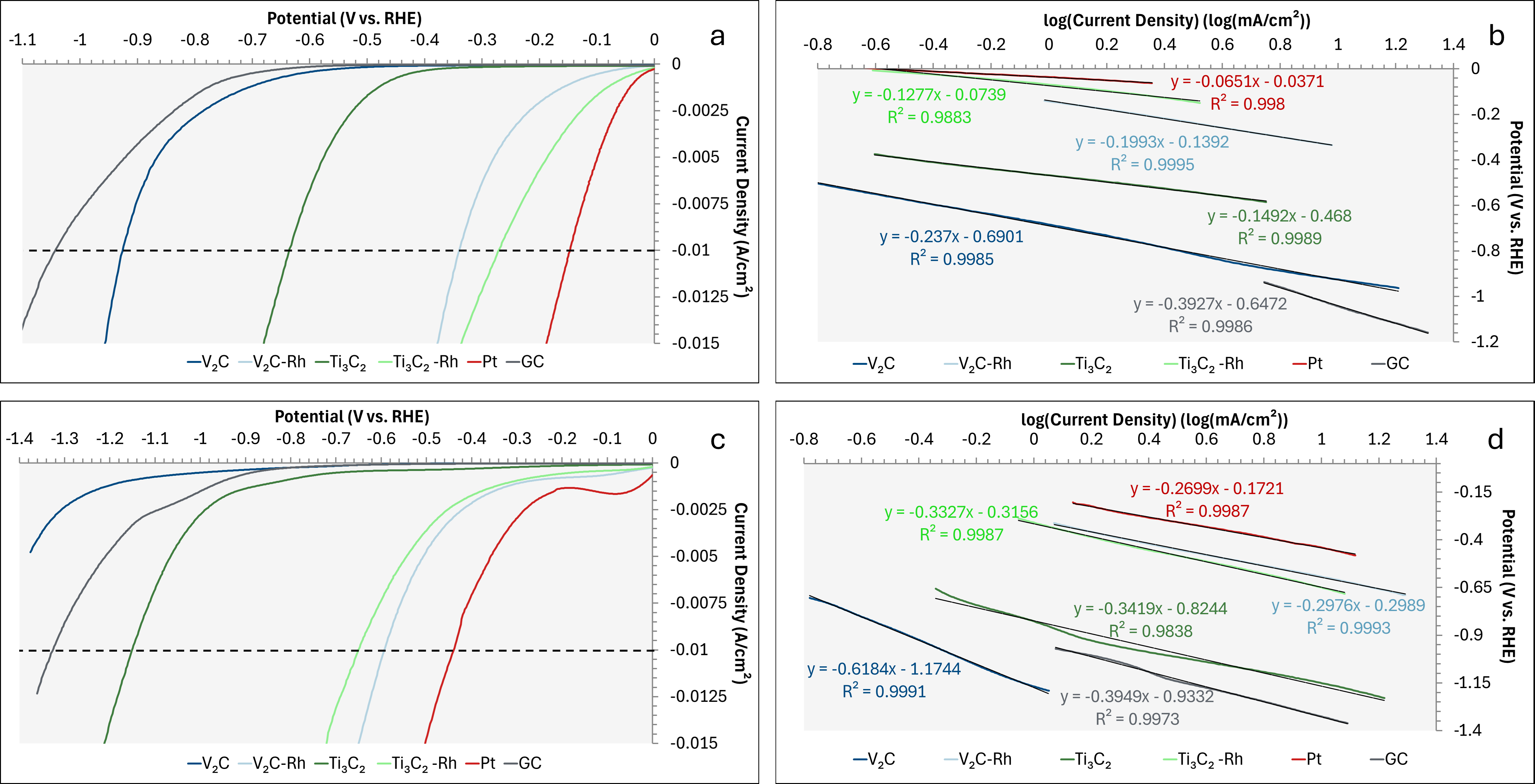


**Figure S5.** Electrochemical evaluation of obtained electrocatalysts and reference materials towards HER. LSV curve in 1M NaOH (a) and PBS (b). Linear fitting of Tafel plots for HER in in 1M NaOH (c) and PBS (d).


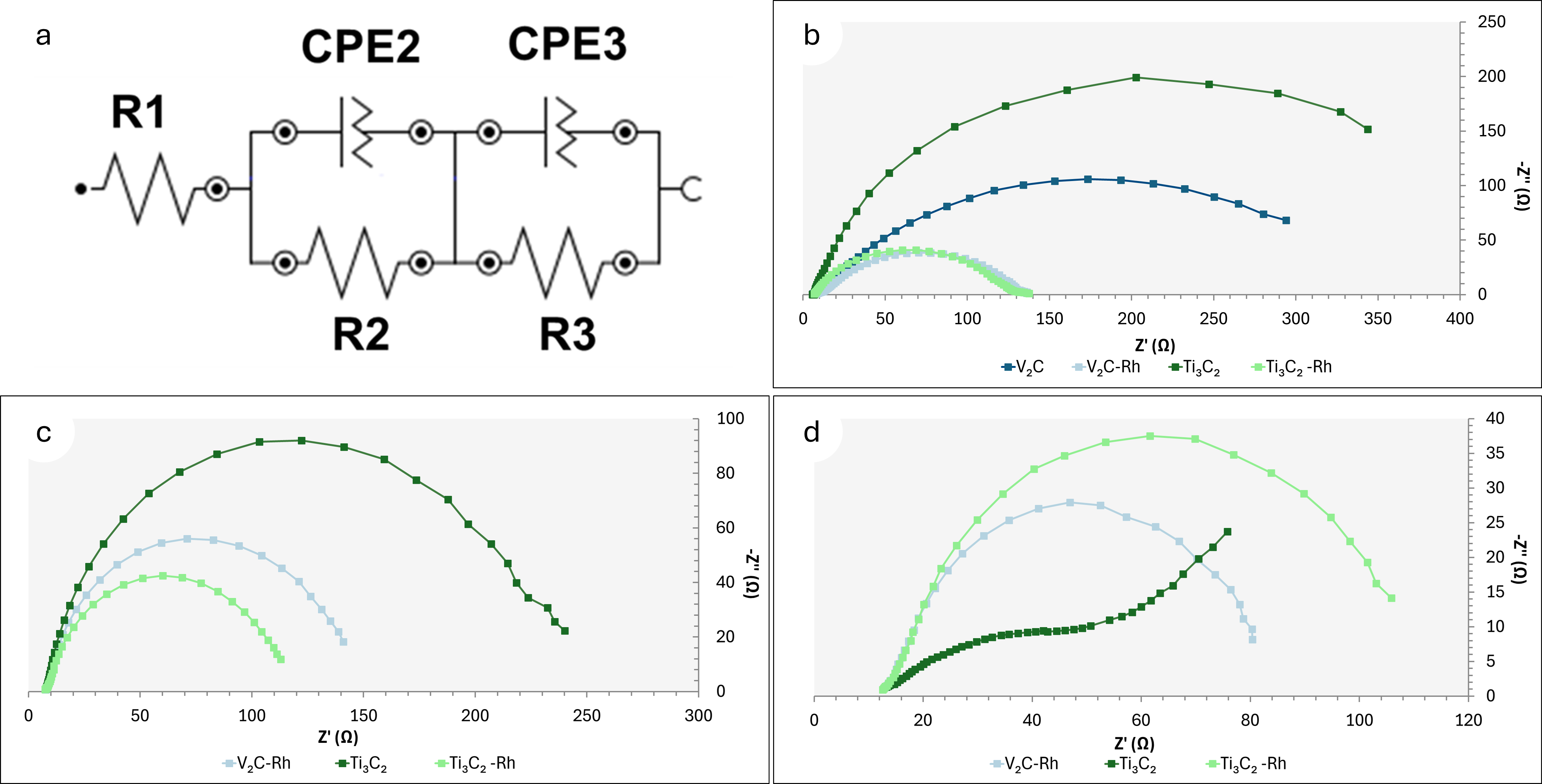


**Figure S6.** The equivalent circuit corresponds to the EIS spectra (a), EIS of the samples in acidic (b), basic (c) and neutral (d) media.


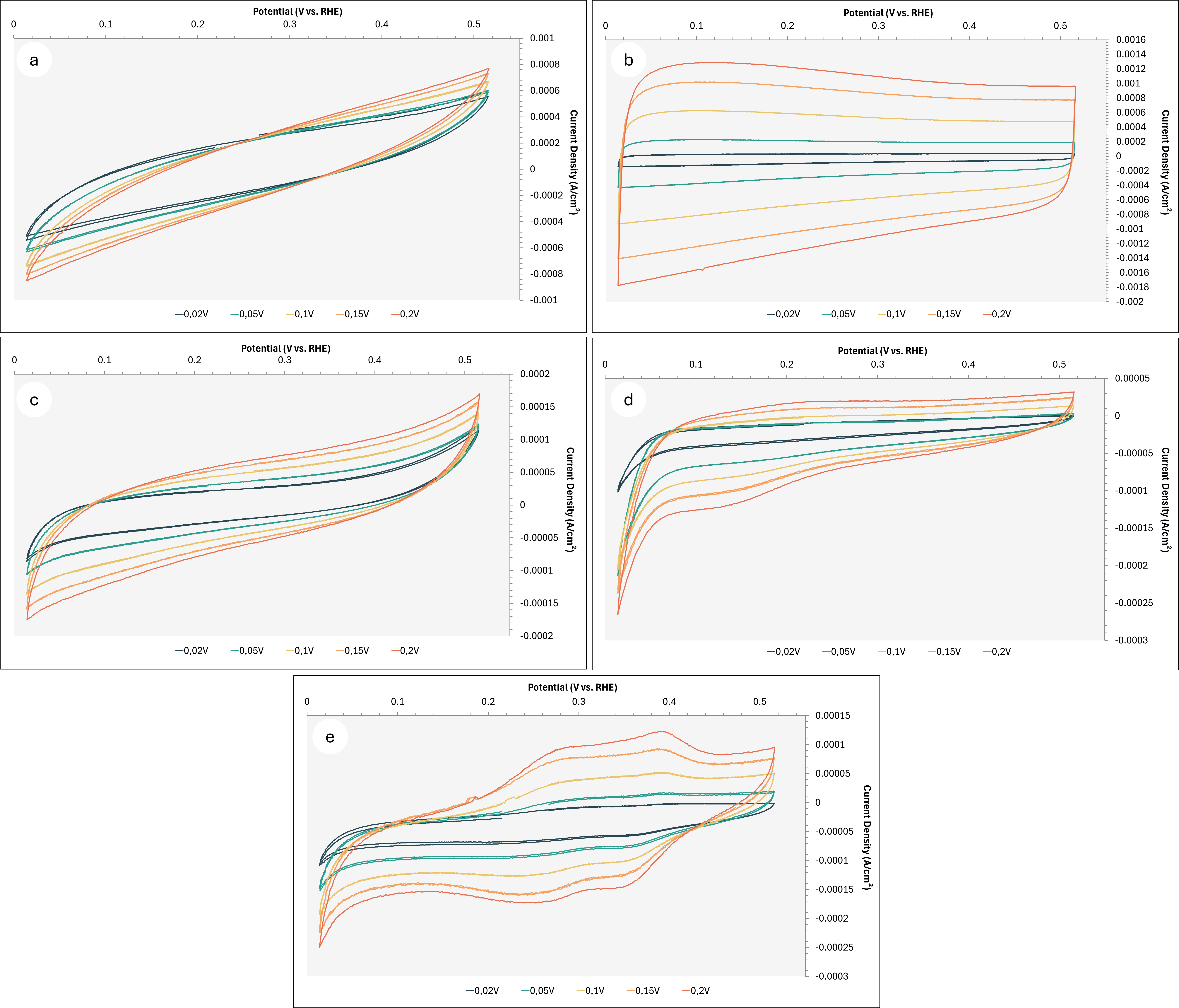


**Figure S7.** Cyclic voltammograms of the V₂C (a), Ti₃C₂(b), V₂C -Rh (c), Ti₃C₂-Rh (d), platinum electrode (e).


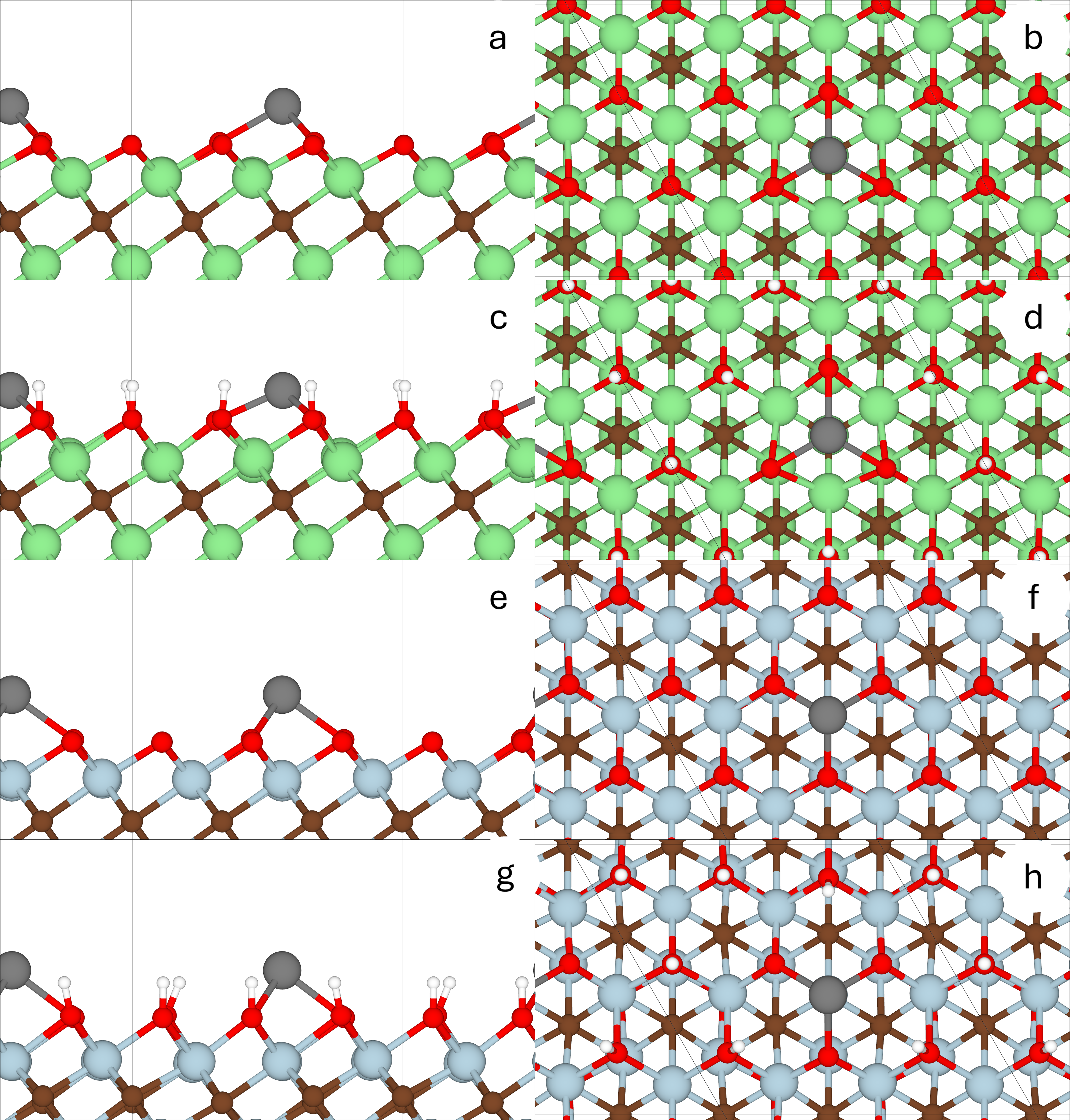


**Figure S8.** Side and top of the Rh site on the nonprotonated (a, b) and protonated (c, d) Ti₃C₂, and nonprotonated (e, f) and protonated (g, h) V₂C. V - blue; Ti - green; C - brown; O - red; H - white; Rh - grey.


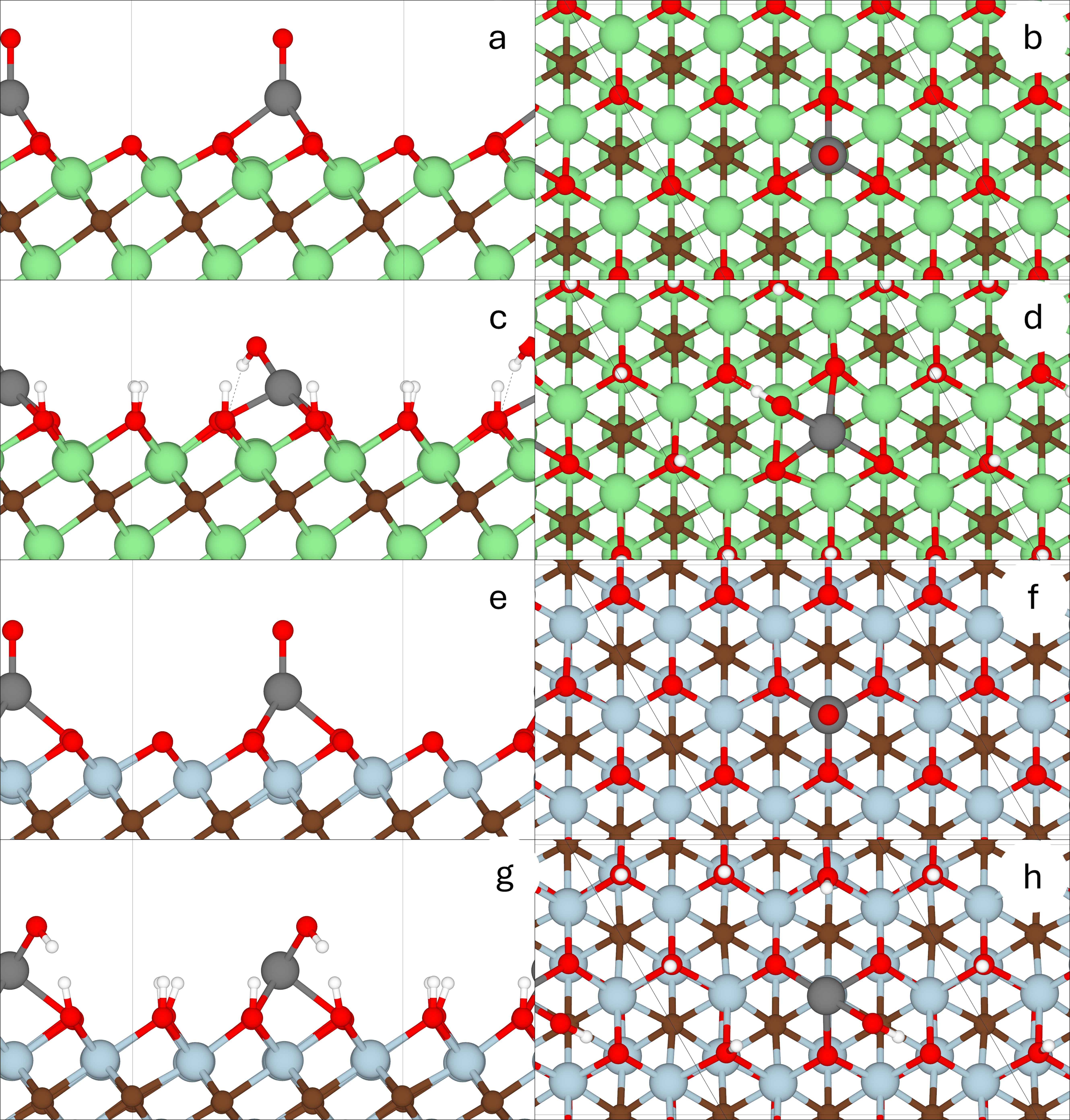


**Figure S9.** Side and top views of the Rh site with one bonded oxygen atom on the nonprotonated (a, b) and protonated (c, d) Ti₃C₂, and nonprotonated (e, f) and protonated (g, h) V₂C. V - blue; Ti - green; C - brown; O - red; H - white; Rh - grey.


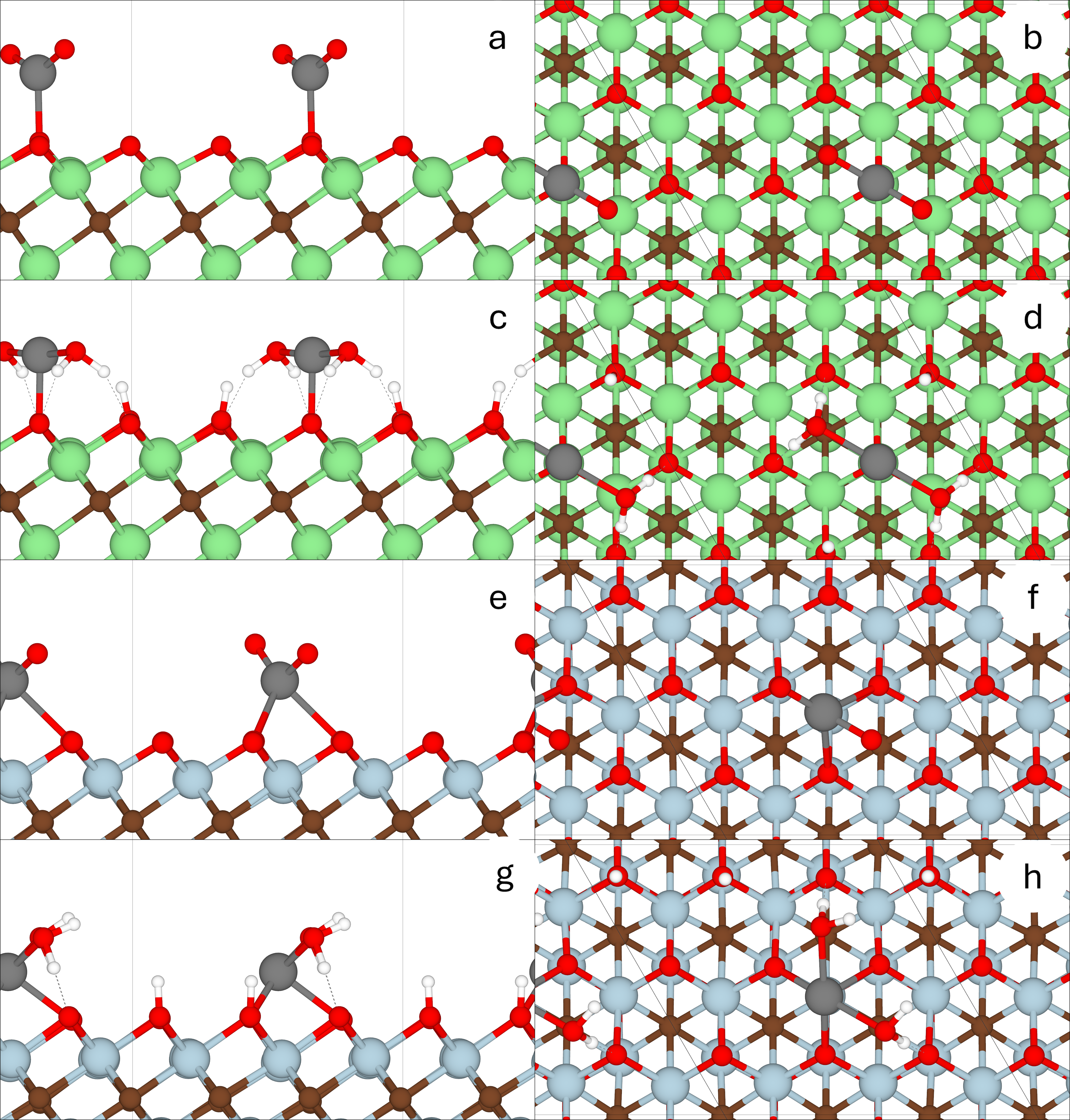


**Figure S10.** Side and top views of the Rh site with two bonded oxygen atoms on the nonprotonated (a, b) and protonated (c, d) Ti₃C₂, and nonprotonated (e, f) and protonated (g, h) V₂C. V - blue; Ti - green; C - brown; O - red; H - white; Rh - grey.


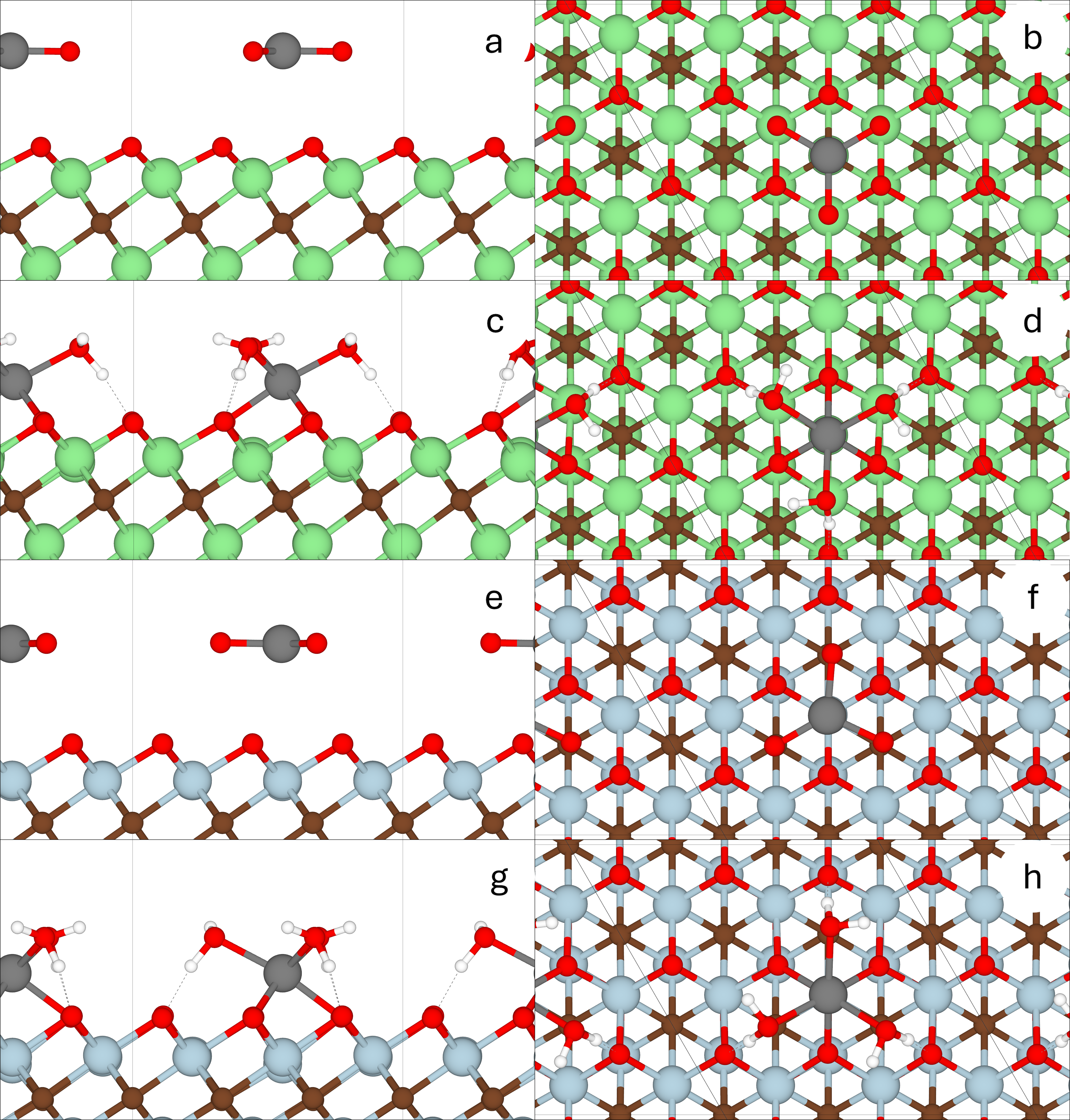


**Figure S11.** Side and top views of the Rh site with three bonded oxygen atoms on the nonprotonated (a, b) and protonated (c, d) Ti₃C₂, and nonprotonated (e, f) and protonated (g, h) V₂C. V - blue; Ti - green; C - brown; O - red; H - white; Rh - grey.

**Table S5.** Comparison of the HER performance of rhodium-decorated MXenes with other single-atom decorated electrocatalysts. ^a^- Rh single atoms on active carbon, ^b,c^ – Rh oxide nanopowders, ^d^ – Rh nanoparticles on carbon, ^e^ – The overpotential at which the turnover frequency was measured, *- this work, ^f^ – Turnover frequencies with and (/) without taking into account average number of layers in MXene nanoflake.

| Material | Solution | η_10_, [mV] | Tafel slope [mv/dec] | Electrode; S, [cm^2^] | Cdl, [mF/cm^2^] | TOF, [s^-1^] | Ref. |
| --- | --- | --- | --- | --- | --- | --- | --- |
| Rh/AC^a^ | 1.0 M NaOH | 48 | 33 | GCE, 0,2 | 16.3 | 1.26 (0.1 V)^e^ | 1 |
| Rh-MoS_2_ | 1.0 M KOH | 18 | 38 | Carbon paper, 2 | 37.67 | 2.34 (0.1V) | 2 |
| Ti₃C₂O_x_:Rh | 0.1 M H_2_SO_4_ | 23 | 27.8 | R-GCE, 0.196 | 101.6 | 14.9 (0.1V) | 3 |
| Ti₃C₂O_x_:Rh | 1.0 M KOH | 29 | 47.6 | R-GCE, 0.196 | 101.6 | - | 3 |
| Ti₃C₂O_x_:Rh | PBS | 85 | 77.4 | R-GCE, 0.196 | 101.6 | - | 3 |
| Ti₃C₂T_x_-NS-Ir | 0.5M H_2_SO_4_ | 57.7 | 50.5 | Carbon paper, 0.25 | 75.5 | 15.7 (0.1V) | 4 |
| Ti₃C₂T_x_-NS-Ru | 0.5M H_2_SO_4_ | 76 | 190 | Carbon paper, 1 | 31 | 0.5 (0.1V) | 5 |
| Ti₃C₂T_x_-Pt | 0.5M H_2_SO_4_ | 38 | 45 | GCE, 0.071 | - | 23.45 (0.1V) | 6 |
| Ti₃C₂T_x_-N-Pt | 0.5M H_2_SO_4_ | 86 | 61 | GCE, 0.071 | - | 5.62 (0.1V) | 6 |
| Ti₃C₂T_x_-Pt | 0.5M H_2_SO_4_ | 34 | 29.4 | GCE 0.196 Vulcan XC-72R | 26.5 | ≈5 (0.1V) | 7 |
| RhO_2_^b^ | 1.0 M KOH | 28 | 33 | Polyimide cloth | - | - | 8 |
| Rh_2_O_3_^c^ | 1.0 M KOH | 261 | 267 | Polyimide cloth | - | - | 8 |
| C/Rh^d^ | 1.0 M KOH | 64 | 63 | Polyimide cloth | - | - | 8 |
| V₂C -Rh | 0.5M H_2_SO_4_ | 128 | 80.6 | GCE, 0.196 | 0.23 | 4.7 (0.1V) | * |
| V₂C -Rh | 1.0 M NaOH | 339 | 199 | GCE, 0.196 | 0.23 | 0.48 (0.1V) | * |
| V₂C -Rh | PBS | 594 | 297.6 | GCE, 0.196 | 0.23 | 4.2 (0.5V) | * |
| Ti₃C₂-Rh | 0.5M H_2_SO_4_ | 127 | 83.8 | GCE, 0.196 | 0.19 | 16.5/4.7^f^ (0.1V) | * |
| Ti₃C₂-Rh | 1.0 M NaOH | 274 | 127 | GCE, 0.196 | 0.19 | 5.51/1.57 ^f^ (0.1V) | * |
| Ti₃C₂-Rh | PBS | 652 | 332.7 | GCE, 0.196 | 0.19 | 11.4/3.4 ^f^ (0.5V) | * |
| Pt | 0.5M H_2_SO_4_ | 43 | 32.4 | - | 0.58 | 16.5 (0.1 V) | * |
| Pt | 1.0M NaOH | 147 | 64.1 | - | 0.58 | 0.53 (0.1V) | * |
| Pt | PBS | 440 | 269.9 | - | 0.58 | 2.5 (0.5V) | * |

References

1. Yu, Z., Xu, J., Feng, S., et al, "Rhodium single-atom catalysts with enhanced electrocatalytic hydrogen evolution performance." *New Journal of Chemistry* 45, no. 13 (2021): 5770-5774. <https://doi.org/10.1039/D1NJ00210D>

2. Yu, F.Y., Sun, H., Tan, H.Q., Li, Y.G., Lee, S.T. and Kang, Z.H., "Dynamic evolution of single-atom Rh site and MoS2 support for hydrogen evolution." *Chem Catalysis* 4, no. 6 (2024). <https://doi.org/10.1016/j.checat.2024.101002>

3. Peng, X., Mi, Y., Liu, X., Sun, J., Qiu, Y., Zhang, S., Ke, X., Wang, X. and Luo, J., 2022. Self-driven dual hydrogen production system based on a bifunctional single-atomic Rh catalyst. *Journal of Materials Chemistry A*, 10(11), pp.6134-6145. <https://doi.org/10.1039/D1TA07375C>

4. Lin, W., Lu, Y.R., Peng, W., Luo, M., Chan, T.S. and Tan, Y., "Atomic bridging modulation of Ir–N, S co-doped MXene for accelerating hydrogen evolution." *Journal of Materials Chemistry A* 10, no. 18 (2022): 9878-9885. <https://doi.org/10.1039/D2TA00550F>

5. Ramalingam, V., Varadhan, P., Fu, H.C., Kim, H., Zhang, D., Chen, S., Song, L., Ma, D., Wang, Y., Alshareef, H.N. and He, J.H., "Heteroatom‐mediated interactions between ruthenium single atoms and an MXene support for efficient hydrogen evolution." *Advanced Materials* 31, no. 48 (2019): 1903841. <https://doi.org/10.1002/adma.201903841>

6. Zhang, J., Wang, E., Cui, S., Yang, S., Zou, X. and Gong, Y., "Single-Atom Pt Anchored on Oxygen Vacancy of Monolayer Ti₃C₂T x for Superior Hydrogen Evolution." *Nano Letters* 22, no. 3 (2022): 1398-1405. <https://doi.org/10.1021/acs.nanolett.1c04809>

7. Wu, Y., Wei, W., Yu, R., et al., "Anchoring sub‐nanometer Pt clusters on crumpled paper‐like MXene enables high hydrogen evolution mass activity." *Advanced Functional Materials* 32, no. 17 (2022): 2110910. https://doi.org/10.1002/adfm.202110910

8. Li, Z., Feng, Y., Liang, Y.L., Cheng, C.Q., Dong, C.K., Liu, H. and Du, X.W., "Stable rhodium (IV) oxide for alkaline hydrogen evolution reaction." *Advanced Materials* 32, no. 25 (2020): 1908521. <https://doi.org/10.1002/adma.201908521>
